# Proteomic analysis reveals co-ordinated alterations in protein synthesis and degradation pathways in LRRK2 knockout mice

**DOI:** 10.1093/hmg/ddy232

**Published:** 2018-06-18

**Authors:** Laura Pellegrini, David N Hauser, Yan Li, Adamantios Mamais, Alexandra Beilina, Ravindran Kumaran, Andrea Wetzel, Jonathon Nixon-Abell, George Heaton, Iakov Rudenko, Mor Alkaslasi, Natalie Ivanina, Heather L Melrose, Mark R Cookson, Kirsten Harvey

**Affiliations:** 1 Cell Biology and Gene Expression Section, Laboratory of Neurogenetics, National Institute of Aging, National Institutes of Health, Bethesda, MD, USA; 2 Department of Pharmacology, UCL School of Pharmacy, University College London, London, UK; 3 Mass-spetrometry Facility, National Institute of Neurological Diseases and Stroke, National Institutes of Health, Bethesda, MD, USA; 4 Neurogenetics Branch, National Institute of Neurological Disorders and Stroke – National Institutes of Health, Bethesda, MD, USA; 5 Department of Neurology, SUNY at Stony Brook, Health Science Center, Stony Brook, NY, USA; 6 Department of Neuroscience, Mayo Clinic Jacksonville, Jacksonville, FL, USA

## Abstract

Mutations in leucine-rich repeat kinase 2 (*LRRK2*) segregate with familial Parkinson’s disease (PD) and genetic variation around *LRRK2* contributes to risk of sporadic disease. Although knockout (KO) of *Lrrk2* or knock-in of pathogenic mutations into the mouse germline does not result in a PD phenotype, several defects have been reported in the kidneys of *Lrrk2* KO mice. To understand LRRK2 function *in vivo*, we used an unbiased approach to determine which protein pathways are affected in LRRK2 KO kidneys. We nominated changes in cytoskeletal-associated proteins, lysosomal proteases, proteins involved in vesicular trafficking and in control of protein translation. Changes were not seen in mice expressing the pathogenic G2019S *LRRK2* mutation. Using cultured epithelial kidney cells, we replicated the accumulation of lysosomal proteases and demonstrated changes in subcellular distribution of the cation-independent mannose-6-phosphate receptor. These results show that loss of LRRK2 leads to co-ordinated responses in protein translation and trafficking and argue against a dominant negative role for the G2019S mutation.

## Introduction

Variation in the Leucine-rich repeat kinase 2 (*LRRK2*) gene account for a proportion of the genetic risk of Parkinson’s disease (PD). Causative mutations in the LRRK2 gene occur in multiple families (1–4) and genome-wide association studies nominated the *LRRK2* locus as contributing to sporadic PD risk (5–7). 

*LRRK2* encodes a large protein containing kinase and GTPase enzymatic regions, which contain pathogenic mutations, surrounded by multiple protein–protein interaction domains (8). Pathogenic mutants are more active than wild-type (WT) LRRK2 *in vitro* and in cellular assays (9–11). As homozygous and heterozygous patients carrying the G2019S mutation have similar risk of developing PD and similar disease progression (12–14), G2019S is a true dominant allele. Potential mechanisms include a gain of normal function, a neomorphic function not seen in the WT protein or a dominant negative effect. Distinguishing whether mutant LRRK2 is pathogenic via higher or lower activity is critically important as kinase inhibitors are being developed as therapeutic agents for PD (15). 

LRRK2 is implicated in vesicular trafficking (16–19), perhaps owing to phosphorylation of Rab GTPases, key regulators in membrane trafficking (9,20). LRRK2 is also involved in the regulation of cytoskeletal dynamics (21–24), including an interaction with β-tubulin isoforms (21). Finally, LRRK2 has been proposed to influence protein translation (25–28) although the precise mechanism(s) involved have not been delineated. Whether the proposed effects on protein translation are related to vesicular and/or cytoskeletal events are uncertain.

Transgenic or knockout (KO) mouse models do not consistently recapitulate the primary PD pathologies, including loss of dopamine neurons in the *substantia nigra* (18,29–31). However, multiple groups have reported that loss of LRRK2 causes age-dependent pathological alterations in kidneys (18,29,30,32), potentially owing to high expression of *Lrrk2* relative to *Lrrk1* (30). LRRK2-KO kidneys have altered texture, an increase in apoptotic cell death and inflammation (18), and an accumulation of lipofuscin (18,29,30,32) that may occur owing to altered lysosomal function (18,29). Some of LRRK2-KO phenotypes are reproduced in kinase dead LRRK2 knock-in mice or mice exposed to kinase inhibitors, but not in kidneys from homozygous G2019S knock-in mice (29). 

Collectively, these observations suggest that LRRK2-KO kidneys can be used to investigate the normal function of this protein. Here, we performed two proteomic screens comparing LRRK2 deficient or LRRK2 G2019S knock-in mouse kidneys with age-matched controls to address endogenous LRRK2 function *in vivo*. Our findings demonstrate that, first, LRRK2 deficiency results in proteome changes showing enrichment in proteins involved in cytoskeletal stability, lysosomal degradation and protein translation and, secondly, that the G2019S mutant does not mimic the KO state. Therefore, loss of LRRK2 influences multiple cellular pathways in a co-ordinated manner and G2019S does not result in overlapping proteomic changes.

## Results

### Differentially expressed vesicular trafficking and protein translation proteins in LRRK2-KO kidneys

To survey proteomic differences between LRRK2-KO mice and WT littermate controls, we performed iTRAQ with ultracentrifugated fractions, a 10K supernatant enriched in cytosolic proteins and a 10K pellet enriched in microsomal proteins. This fractionation step was performed with the intent to reduce sample complexity and improve peptide coverage in our iTRAQ screen. We focused on changes in protein abundance as a measure of differential expression to provide an unbiased and quantitative insight into physiological processes such as protein synthesis and degradation potentially affected by LRRK2.

**Figure 1.**
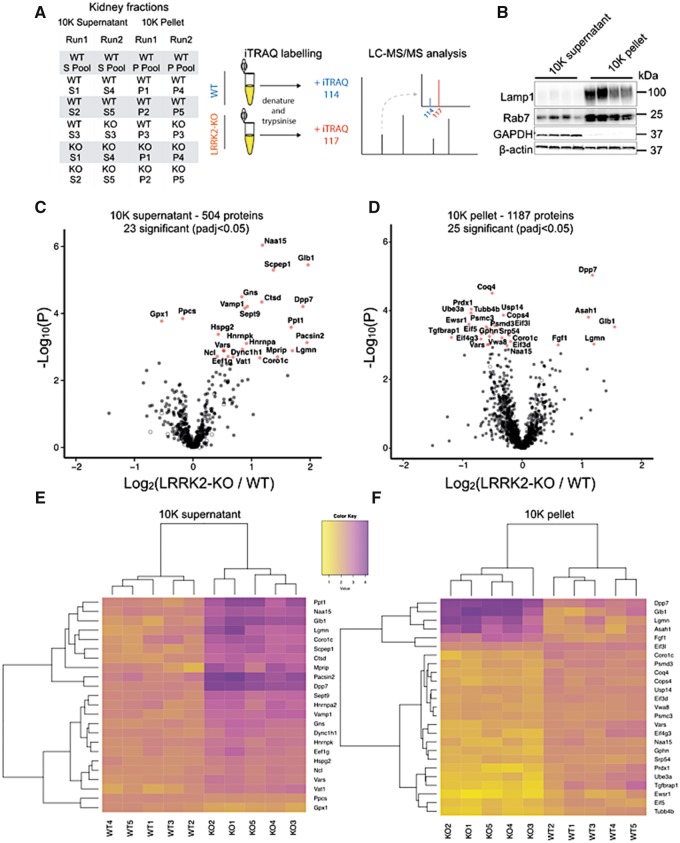
** **Proteomic analysis of LRRK2 KO kidneys. (**A**) Tables of the iTRAQ runs: 10K supernatants and 10K pellets for two independent experiments. Schematic experimental plan including sample preparation, representative iTRAQ labeling, peptide fractionation via LC followed by tandem MS. (**B**) Immunoblots from kidney enriched fractions for endo-lysosomal markers Rab7 and LAMP1, and for the cytosolic marker GAPDH (*n* = 4). Volcano plots of the 504 proteins quantified in the 10K supernatants (**C**) and of the 1187 proteins quantified in the 10K pellets (**D**) (*n* = 5, Welch *T*-test, *P* values corrected using Benjamini–Hochberg post hoc test). Heat maps of significant genes differently regulated in 10K supernatants (**E**) and 10K pellets (**F**). WT, wild-type; KO, LRRK2-KO; S, 10K supernatant; P, 10K pellet.

We compared each fraction from each animal against a common reference pool, with both genotypes distributed across different iTRAQ runs (Fig. 1A). To confirm sample enrichment, we performed immunoblots to detect the abundance of specific protein markers for each fraction. As expected, late-endosome and lysosome markers, the small GTPase Rab7 and the membrane protein LAMP1, were significantly enriched in the 10K pellets compared with the 10K supernatants (Fig. 1B), although Rab7 was also detected in 10K supernatants. In addition, the cytosolic protein GAPDH was enriched in the 10K supernatant (Fig. 1B). As the goal of this enrichment was to improve protein detection rather than the generation of more defined pure fractions, these samples were used for the following proteomic experiments.

**Figure 2.**
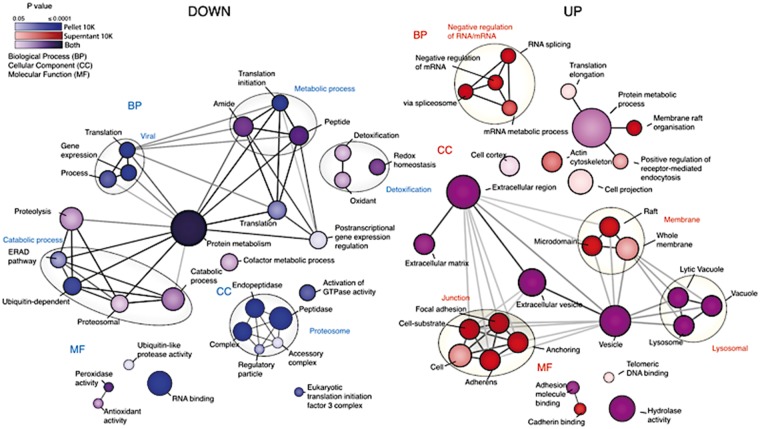
Altered gene ontology pathways in LRRK2 KO kidneys. Map of dysregulated gene ontology pathways was generated using the Enrichment map Cytoscape plug-in. Analysis was performed with gProfileR package using a false discovery rate correction. Cellular component (CC), biological process and molecular function have been depicted for each gene set. Node size corresponds to the number of genes in each GO term. Only GO terms ranging between 10 and 5000 were included in this analysis. Node color corresponds to which gene set each enrichment is derived from with shading being proportional to *P* value (cutoff, ≤0.05). Edges signify significant overlap between GO terms using a cutoff of 0.5 similarity coefficient, darker edges indicate greater overlap between terms.

In the 10K supernatants samples, we identified 700 (Run 1) and 600 (Run 2) unique proteins, with 504 common proteins. In the 10K pellets, 1500 (Run 1) and 1800 (Run 2) unique proteins were detected, with 1187 shared hits. Totally, 375 of the proteins were detected in both fractions. In the 10K supernatants, 23 proteins differed significantly in abundance between LRRK2-KO and controls, with most proteins showing higher detection in the KO animals compared with WT (Fig. 1C). We further identified 25 significantly different proteins in the 10K pellets, of which 5 were also significantly different in the 10K supernatants (Fig. 1D). Unsupervised hierarchical clustering of differential proteins separated genotypes in both 10K supernatants and 10K pellets (Fig. 1E and F). We classified differentially abundant proteins using gene ontogeny (GO) analysis (Fig. 2). Consistent with previous findings (18), we found significant enrichment in categories related to protein degradation (Fig. 2), including the GO: cellular compartment terms lytic vacuole and lysosomal lumen (Fig. 2; Supplementary Material, Fig. S1B). These categories were populated by multiple lysosomal enzymes including cathepsin D (Ctsd), legumain (Lgmn), dipeptidyl peptidase 7 (Dpp7) and galactosidase beta 1 (Glb1), all found in higher abundance in LRRK2-KO compared with WT mice (Fig. 1C and D).

Cytoskeletal proteins were also differentially expressed in KO kidneys. Lower levels of Tubb4b and gephyrin (Gphn) were noted in LRRK2-KO 10K pellets (Fig. 1D and F), while coronin 1C (Coro1c), protein kinase C and casein kinase substrate in neurons protein 1 (Pacsin2) and septin 9 (Sept9), were significantly higher in the LRRK2-KO 10K supernatants compared with controls (Fig. 1C and E). Many of these proteins were also represented in the GO: Cellular compartment term ‘extracellular exosome’ (*P* = 3.13 ×10^−12^ (Fig. 2; Supplementary Material, Fig. S1B).

A third set of proteins related to ‘initiation of mRNA and protein translation’ was differentially abundant in LRRK2-KO 10K pellets (Fig. 2; Supplementary Material, Fig. S1D). Among these, heterogeneous nuclear ribonucleoprotein K (Hnrnpk) and elongation factor 1-gamma (Eef1g) were higher in the 10K supernatants of KO animals compared with controls, while the eukaryotic translation initiation factor 4 subunit G3 (Eif4g3) and related proteins Eif5, Eif3l were lower in KO 10K pellets.

In summary, we found clear significant differences in abundance of proteins functioning in synthesis and catabolic function. Most of these proteins belonged to three main categories, proteins important for protein translation, cytoskeletal function and lysosomal function.

### G2019S knock-in mutant kidneys do not show detectable differences in protein expression levels

**Figure 3.**
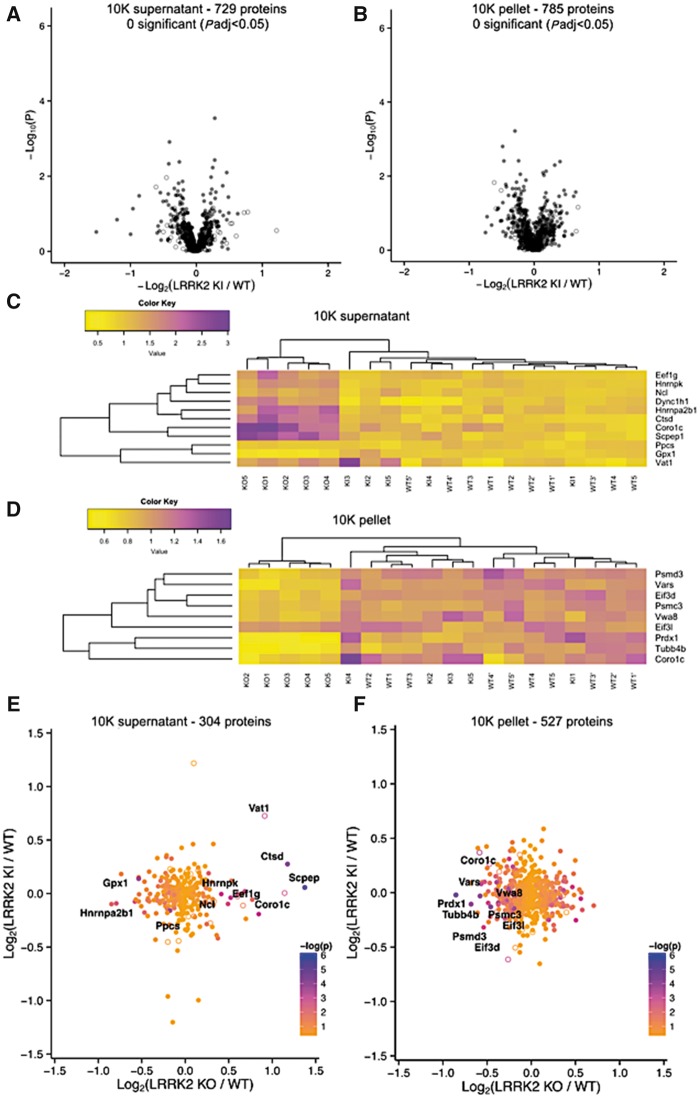
Proteomic analysis of LRRK2 G2019S knock-in kidneys. Volcano plots of the 729 proteins quantified in the 10K supernatants (**A**) and of the 785 proteins quantified in the 10K pellets (**B**) (*n* = 5, Welch *T*-test, *P* values corrected using Benjamini–Hochberg post hoc test). Heat maps of detected genes (with *P* value <0.05 before correction) in 10K supernatants (**C**) and 10K pellets (**D**). Correlation of fold-changes between G2019S-KI and LRRK2-KO, in 10K supernatants (**E**) and 10K pellets (**F**).

Collectively, the above data nominate a series of proteins that respond to LRRK2 deficiency *in vivo*. To address effects of a PD associated *Lrrk2* mutation, we performed a second iTRAQ experiment using LRRK2-G2019S knock-in animals. We identified 729 unique proteins in the 10K supernatants (Fig. 3A) and 785 unique proteins in the 10K pellets (Fig. 3B). However, we did not detect any significant differences in protein abundance between genotypes in either fraction (Fig. 3A and B). Given that the number of proteins detected in each experiment varied and that iTRAQ ratios have a compressed dynamic range (33), we were concerned that the apparent negative result in the G2019S experiment was owing to an underestimate of true differences. We therefore directly compared the KO and G2019S experiments for those proteins detected in all experiments for each fraction. Using hierarchical clustering, we found that the KO samples separated from both G2019S knock-in and WT samples but knock-in and WT did not (Fig. 3C and D). No correlation was found between LRRK2-G2019S knock-in protein abundance in comparison to WT for 10K supernatants (Fig. 3E; Pearson’s correlation between log2-fold differences, *R* = 0.0496, *P *=* *0.389, *n* = 304 proteins) or pellets (Fig. 3F; Pearson’s correlation between log2-fold differences, *R* =-0.0445, *P *=* *0.308, *n* = 527 proteins).

We considered whether the lack of effect of the G2019S genotype might result from temporal separation of experiments. To address this, we re-ran KO 10K supernatants against WT controls as in the first iTRAQ experiment. Again, we saw similar, significant differences in genotype (Supplementary Material, Fig. S2A) sufficient to separate genotypes using hierarchical clustering (Supplementary Material, Fig. S2B). Moreover, there was a positive correlation between the Log2 ratios of KO to WT proteins in the two runs (Supplementary Material, Fig. S2C) but again not with the G2019S knock-in proteins (Supplementary Material, Fig. S2D). Overall, these results show that the G2019S allele has no distinguishable effect on the proteome compared with the strong effect on protein abundance observed upon *Lrrk2* KO under the used experimental conditions.

### Validation of iTRAQ results confirms deregulation of proteins important for translational, lysosomal and cytoskeletal function

**Figure 4.**
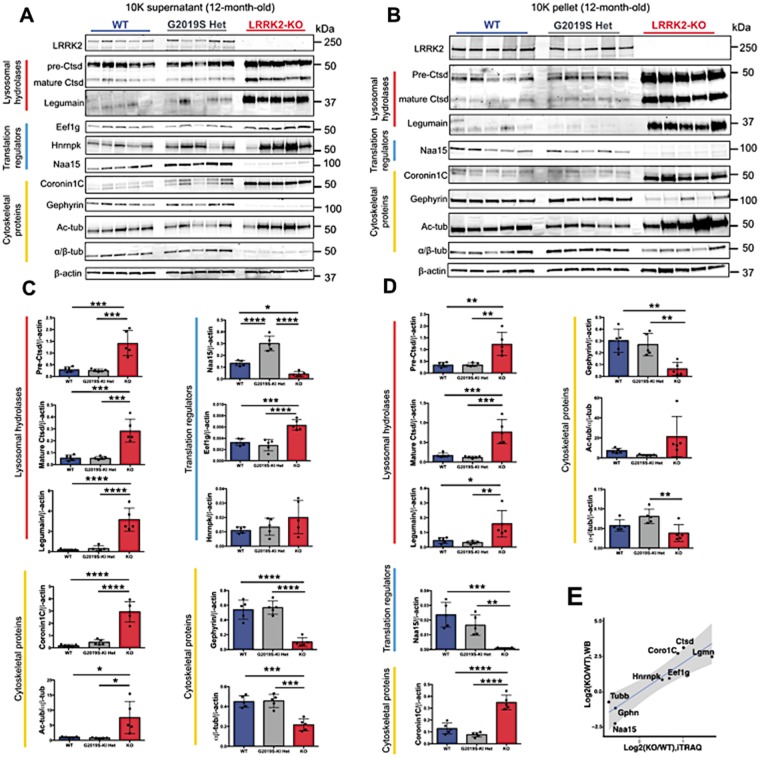
Validation of iTRAQ proteomics results. (**A**) Immunoblots for LRRK2 and the iTRAQ hits of interest normalized for the loading control β-actin in the 10K supernatants and in the 10K pellets (**B**). Quantifications of immunoblots from cytsolic (**C**) and microsomal (**D**) enriched fractions. (**E**) Comparison of the log2-fold expression (KO/WT) using the iTRAQ data compared with the immunoblot data.

To validate these results, immunoblots were performed for candidate proteins using the same protein extracts from the initial 12-month-old cohort of animals (*n* = 5 per group) and G2019S knock-in animals for direct comparison to LRRK2-KO kidneys (Fig. 4A–E). We first confirmed the expected absence of LRRK2 in both LRRK2-KO enriched fractions (Fig. 4A and B). In our proteomic analysis, cathepsin D was significantly higher in the LRRK2-KO 10K supernatants compared with controls. Immunoblot results confirmed higher levels for both mature [*P* = 0.0001, Bonferroni post hoc test from one-way analysis of variance (ANOVA), *n* = 5 animals] and precursor (*P* = 0.0004) forms of cathepsin D in LRRK2-KO (Fig. 4A and C). There were no differences between WT and LRRK2-G2019S for cathepsin D (Fig. 4A and C). Similarly, higher levels of the lysosomal protease legumain were confirmed in both fractions by immunoblot (*P* < 0.0001; Fig. 4A and C). Legumain was not detected in the G2019S iTRAQ screen, but immunoblot of the LRRK2-G2019S enriched fractions did not detect any difference in legumain expression compared with controls (Fig. 4A, C and D). Cathepsin D mature (*P* = 0.0006) and precursor forms (*P* = 0.0014) were also higher in the LRRK2-KO, but not in LRRK2-G2019S, 10K pellets compared with WT controls (Fig. 4B and D). Legumain was nominated as increased in LRRK2-KO 10K pellets by iTRAQ (*P* adjusted =0.049), which was confirmed using immunoblotting in KO samples (*P* < 0.05) but not in G2019S knock-in samples (Fig. 4B and D).

Our iTRAQ results indicated that NatA Auxiliary Subunit 15 (Naa15), a protein important for co-translational protein acetylation that may also interact with the cytoskeleton (34), was lower in LRRK2-KO 10K pellets and higher in LRRK2-KO 10K supernatants compared with controls (Fig. 1B and C). We confirmed lower protein levels of Naa15 in 10K pellets (*P* = 0.0003) (Fig. 4B and D) but found that Naa15 expression was also lower in 10K supernatants (*P* = 0.011) (Fig. 4A and C). Naa15 protein expression was significantly increased in G2019S 10K supernatants, compared with LRRK2-KO or WT samples (*P* < 0.0001) (Fig. 4A and C). Two additional proteins with importance for protein translation, elongation factor 1-gamma (Eef1g) and heterogeneous nuclear ribonucleoprotein K (Hnrnpk), both showed a significant increase in LRRK2-KO but no change in G2019S knock-in tissue (Fig. 4A and C).

We next examined a series of cytoskeleton-associated proteins. The actin binding protein coronin 1C (Coro1c) was detected as more abundant in LRRK2-KO mice compared with controls in our iTRAQ experiments. A 53 kDa band corresponding to coronin 1C was decreases in the LRRK2-KO kidneys but a lower molecular weight band (37 kDa) was significantly increased by LRRK2 deficiency (*P* < 0.001) (Fig. 3A and D). We therefore infer that the lower band is likely to correspond to the unique peptide detected as increased in the iTRAQ dataset. The microtubule-associated protein gephyrin (Gphn), significantly decreased in LRRK2-KO 10K pellets in iTRAQ, was validated in 10K supernatants (*P* < 0.0001) and pellets (*P* = 0.0025) (Fig. 4A–D). To address the observed changes in tubulin associated proteins including Tubb4b further and given the lack of specific reliable anti-Tubb4b antibodies, we investigated differences in total and acetylated tubulin in our samples. A significant increase in acetylated-tubulin adjusted to α/β-tubulin was detected in 10K supernatants from KO animals compared with WT (*P* = 0.0171). Additionally, α/β-tubulin, normalized expression levels were significantly lower in LRRK2-KO 10K supernatants compared with controls (*P* = 0.0002) (Fig. 4A–D). No significant difference in gephyrin, acetylated or total tubulin was detected between G2019S and WT animals.

To investigate whether changes could be observed in neural tissue, immunoblots were performed on cerebral cortex, hippocampus and striatum from the same 12-month-old mice. No significant difference was detectable between LRRK2-KO and WT mice for most of the proteins tested (Supplementary Material, Fig. S3) with the exception of gephyrin, where expression levels were lower in LRRK2-KO hippocampus (*P* < 0.05, *t*-test, *n* = 5 animals) but no difference was shown in other brain regions tested (Supplementary Material, Fig. S3E and F). Therefore, the effects of LRRK2 deficiency on protein expression levels in the brain are more modest than those in the kidney.

These results validate 11 out of the 12 nominated protein expression differences between LRRK2-KO and WT kidneys, with the exception being Naa15 in the 10K supernatants. Overall, we found that fold differences between genotypes were larger using immunoblotting than with iTRAQ (Fig. 4E), consistent with the issue of ratio compression in iTRAQ experiments (33). We did not detect differences between WT and G2019S knock-in animals apart from Naa15, which was more abundant in the 10K supernatants, in contrast to KO lysates.

### Additional biological validation reveals age-dependent changes in LRRK2-KO kidneys

Alterations in LRRK2-KO kidneys show age-dependent effects (18). To test whether this was true for all candidate proteins we examined a series of independent cohorts of LRRK2-KO animals at different ages. Given that we were able to detect our proteins of interest by immunoblot without enrichment and we did not observe significant protein migration between enriched fractions, we performed immunoblots on total kidney homogenates to avoid any possible protein loss.

**Figure 5.**
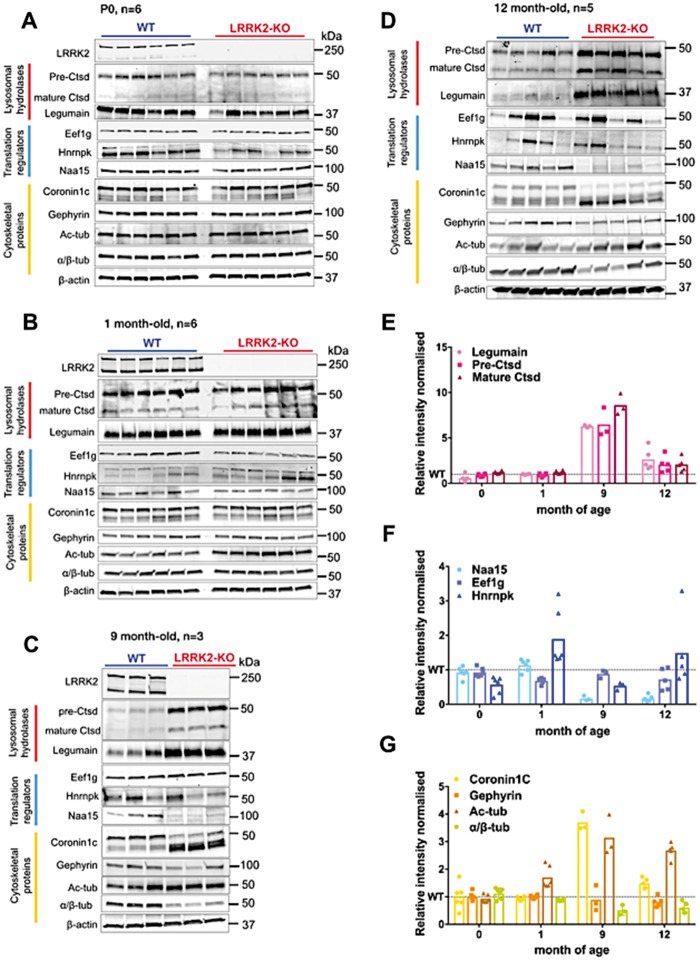
Biological validation of iTRAQ results across aging cohorts. Immunoblots for the iTRAQ proteins of interest using kidney homogenates from P0 (**A**), 1-month-old (**B**), 9-month-old (**C**), 12-month-old (**D**) LRRK2-KO and control mice. Plots indicating the differences in the iTRAQ candidate lysosomal hydrolases (**E**), translational regulators (**F**) and cytoskeletal-associated proteins (**G**) quantified from the immunoblots, between aging cohorts (Supplementary figure for additional immunoblot quantification graphs).

No significant differences were observed in kidney lysates from LRRK2-KO and control newborn (P0) mice (*n *=* *6 animals per genotype) in any of the markers examined except for a significant decrease in Hnrnpk levels in LRRK2-KO kidneys (Fig. 5A and F;Supplementary Material, Fig. S4). In 1-month-old animals (*n *=* *6), we observed a small but significant accumulation of the mature form of cathepsin D, an equally small but significant increase in Hnrnpk and decrease in Eef1g, and significantly lower levels of α/β-tubulin and a substantial increase in acetylated-tubulin in KO animals compared with WT. No changes were detected for legumain, Naa15, coronin 1C, gephyrin (Fig. 5B;Supplementary Material, Fig. S4).

In an independent cohort of 9-month-old mice (*n *=* *3), cathepsin D and legumain levels were significantly higher in LRRK2-KO kidney homogenates compared with WT animals. Naa15 protein levels were significantly decreased in the LRRK2-KO samples and a similar level of coronin 1C was noted as seen in the initial cohort used for proteomics. We also detected significantly lower levels of α/β-tubulin, together with higher levels of tubulin-acetylation (Fig. 5C;Supplementary Material, Fig. S4). We also confirmed similar differences in kidney homogenates from the 12-month-old mouse cohort (*n* = 5) used for iTRAQ experiments (Figs 4 and 5D; Supplementary Material, Fig. S4).

Collectively, these results show that an increase in lysosomal proteases together with a decrease in total tubulin accompanied by an increase in tubulin acetylation are detectable throughout most of the time points and therefore are the most consistent. Changes in translational proteins were found to be the least consistent over time. Of the nominated differences between genotypes, at the earliest ages there were changes in Hnrnpk and Eef1g, accumulation of mature cathepsin D, and loss of α/β-tubulin accompanied by an increase in acetylated tubulin. These results suggest that the primary responses to LRRK2 deficiency in the kidney include translational, lysosomal and cytoskeletal changes but that changes over time affect more consistently in particular lysosomal hydrolases and cytoskeletal proteins.

### Accumulation of endo-lysosomal clusters in LRRK2-KO kidneys

**Figure 6.**
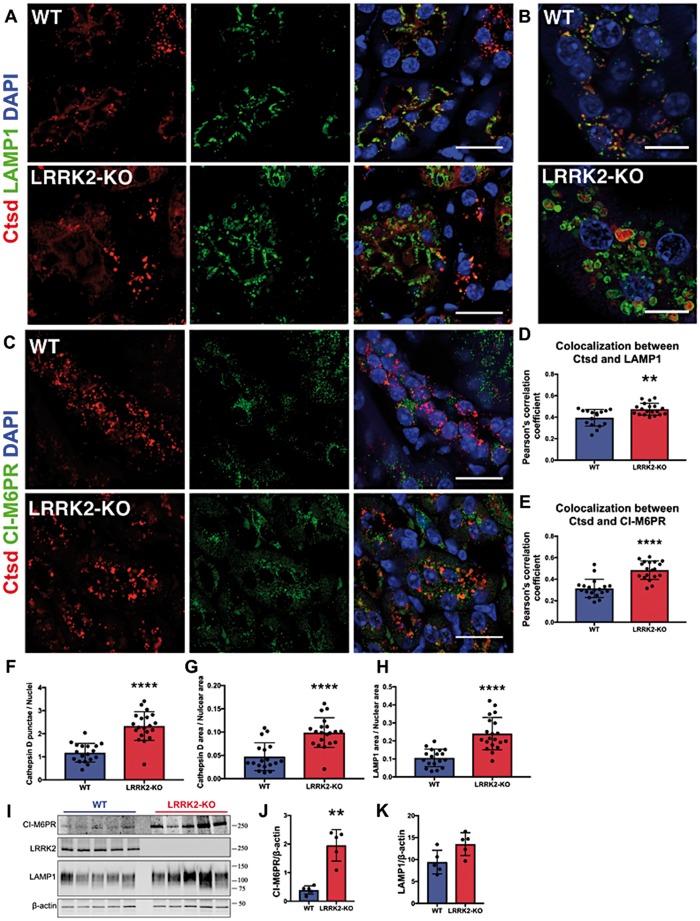
Histological characterization of LRRK2 KO kidneys. (**A**) Confocal imaging of WT, LRRK2-KO (5- to 6-month-old, *n* = 3) for cathepsin D (rabbit polyclonal antibody, AlexaFluor 568 secondary antibody), LAMP1 (rat monoclonal antibody, AlexaFluor 488 secondary antibody) and DAPI staining. Scale bar: 20 μm. (**B**) Representative Airy scan images of the same WT and LRRK2-KO kidneys for cathepsin D, LAMP1 and DAPI staining. Scale bar: 10 μm. (**C**) Confocal imaging of the same WT and LRRK2-KO kidneys for cathepsin D (goat polyclonal antibody, AlexaFluor 568 secondary antibody) and M6PR (rabbit monoclonal antibody, AlexaFluor 488 secondary antibody) and DAPI staining. Scale bar: 20 μm. (**D**) Quantification of cathepsin D and LAMP1 colocalization in the kidney sections shown in panel (A). (**E**) Quantification of cathepsin D and M6PR colocalization in the kidney sections shown in (**C**). Data points represent Pearson’s correlation coefficient from at least five images per animal, with multiple cells per image, *n* = 3 mice per genotype. (**F**) Number of cathepsin D punctae per nuclei and area (μm^2^) of cathepsin D (**G**) and LAMP1 (**H**) positive vesicles. (**I**) Immunoblot analysis of WT and LRRK2-KO kidney lysates (12-month-old total homogenates, *n* = 5) using CI-M6PR and LAMP1 antibodies. Quantifications of CI-M6PR (**J**) and of LAMP1 (**K**) immunoblots normalized for the loading control β-actin.

To further characterize the LRRK2-KO lysosomal phenotype, we performed immunofluorescent staining of kidney sections from 6-month-old animals. As expected, LRRK2-KO kidneys were larger, heavier and darker in color than their WT counterparts (Supplementary Material, Fig. S5A and B). The most prominent observed changes were enlarged and more numerous cathepsin D positive punctae in the proximal tubules of LRRK2-KO kidneys (Fig. 6A, F, G). To better discern cathepsin D positive vesicles, we used super-resolution microscopy (Fig. 6B). Cathepsin D partially colocalized with the late-endosomal/lysosomal marker LAMP1 in WT animals but showed more distinct localization to the lysosomal membrane in KO animals (Fig. 6B). LAMP1 positive structures appeared also significantly enlarged in LRRK2-KO (Fig. 6A and H). Analysis of cathepsin D and LAMP1 show an increase in colocalization between the two markers in LRRK2-KO kidneys indicating an accumulation of cathepesin D in the endo-lysosomal compartment (Fig. 6D).

Lysosomal proteases such as cathepsin D are synthesized in the ER as inactive precursors, recognized by mannose-6-phosphate receptor (M6PR) and transported via the *trans*-Golgi network and sorting endosomes to lysosomes where they undergo maturation by pH-dependent proteolytic cleavage (35). We hypothesized that LRRK2 deficiency affects cathepsin D protein sorting via its receptor. To test this hypothesis, we analyzed cathepsin D and cation-independent M6PR (CI-M6PR) by immunofluorescence in kidney sections (Fig. 6C). We observed an increase in colocalization between cathepsin D and CI-M6PR in LRRK2-KO kidneys (Fig. 6E) suggesting a scenario in which cathepsin D and its receptor are accumulating in an endo-lysosomal compartment and fail to recycle back to the Golgi. To further investigate this hypothesis, we probed 12-month-old kidney extracts for LAMP1 and M6PR by immunoblot. We observed a significant increase in M6PR in LRRK2-KO kidney extracts (Fig. 6I and J). Surprisingly, no significant differences in LAMP1 levels were detected although we can observe an increasing trend in the KO (Fig. 6I and K). Taken together, these results suggest that cathepsin D trafficking is dysregulated in LRRK2 KO kidneys.

### Primary kidney cells show that lysosomal dysregulation is a primary consequence of LRRK2 deficiency

To investigate the possibility of dysregulated cathepsin D trafficking further and gain more in depth inside into the most reproducible and earliest increase in protein abundance observed in our animal cohorts, we cultured epithelial cells from the kidney cortex from WT and LRRK2-KO animals.

**Figure 7.**
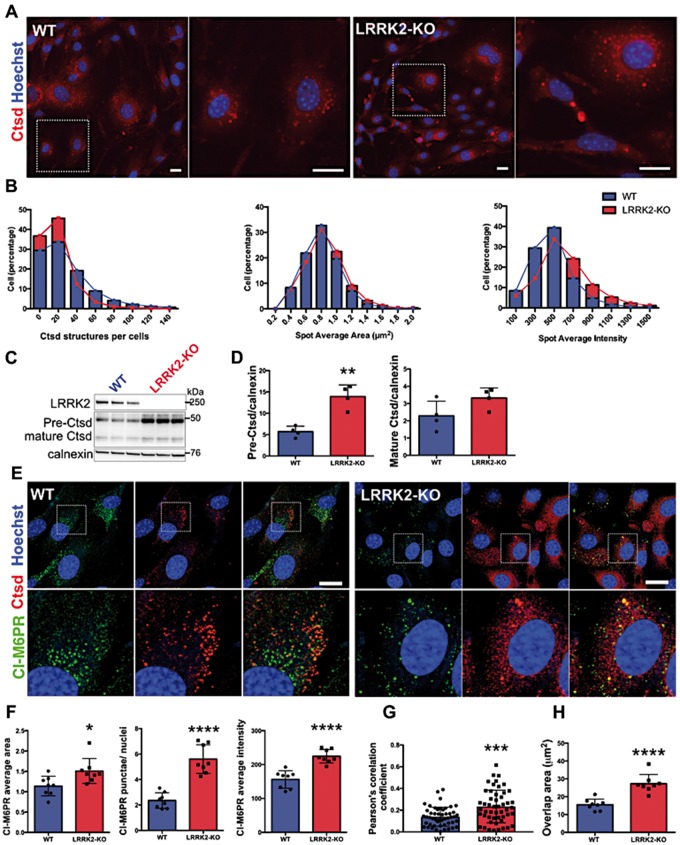
Impaired trafficking of mannose-6-phosphate receptor in LRRK2 KO cells. (**A**) Cellomics representative images of WT and LRRK2-KO primary kidney cells. (**B**) Quantifications of percentage of cells with cathepsin D structures, average spot area (μm^2^) and average intensity counted using the spot detector bioapplication of the Cellomics arrayscan. (**C**) Immunoblots for cathepsin D in total homogenates from LRRK2-KO and control primary kidney cells. (**D**) Immunoblot quantifications of cathepsin D precursor and mature forms. (**E**) Representative confocal images of LRRK2-KO and wild-type primary kidney cells stained in red for cathepsin D (anti-rabbit, AlexaFluor 568 secondary antibody), in green for M6PR (anti-mouse, Alexa 488 secondary antibody). Nuclei in blue were stained with Hoechst. Scale bar=20 μm (5 μm for magnification). (**F**) Quantifications of percentage per cell, average area and average intensity of M6PR punctae in LRRK2-KO and wild-type primary kidney cells. (**G**) Pearson’s correlation coefficient measured by manually quantifying 50 cells after confocal imaging and (**H**) overlap area (μm^2^) measured by high content image analysis.

As *in vivo*, cells lacking LRRK2 had fewer but larger cathepsin D positive clusters compared with WT controls, resulting in an overall accumulation of cathepsin D (Fig. 7A–D). We did not detect a significant difference in LAMP1 fluorescence intensity (Supplementary Material, Fig. S7). This is in contrast to what was observed in kidney histological sections in LRRK2-KO (Fig. 6). The lack of a significant increase in LAMP1 between genotypes in cells is in accordance with cathepsin D increase in precursor but not in the mature form (Fig. 7C). To further investigate this difference we measured the colocalization of cathepsin D with LAMP1 in primary kidney cells (Supplementary Material, Fig. S7). Here, we observed a decrease in colocalization between the two markers in LRRK2-KO cells. These data suggest that in our cell model the lysosomal hydrolase cathepsin D is improperly processed and accumulates in pre-lysosomal compartments.

In our attempt to provide a mechanistic explanation for this accumulation, we next asked whether we could observe the same differences in the CI-M6PR trafficking pathway reported in kidney sections (Fig. 6C). We observed enlarged, and more dispersed CI-M6PR-positive vesicles in primary LRRK2-KO cells compared with WT cells (Fig. 7E and F) suggesting that defects in vesicle trafficking may be important in LRRK2 effects on lysosomal proteases. To further test this hypothesis, we inhibited lysosomal acidification using bafilomycin-A1 (Supplementary Material, Fig. S6A and B). As expected, mature cathepsin D levels were decreased in response to bafilomycin-A1, whereas we observed a decrease in the precursor form of cathepsin D and increase of pre-pro cathepsin D together with an increase in LC3-II. Interestingly, the differences between genotypes were abolished upon bafilomycin treatment, suggesting that trafficking rather than expression of cathepsin D is the critical regulator of cathepsin D levels (Supplementary Material, Fig. S6A and B). Supporting this idea, we further confirmed that cathepsin D mRNA expression does not differ between genotypes (Supplementary Material, Fig. S6C–G). Another possible interpretation of the results obtained after bafilomycin treatment is that we could observe an increase in cathepsin D secretion, previously reported upon treatment with this lysosomal inhibitor (36).

Defects in cathepsin D trafficking have been reported in cells expressing the dominant negative form of Rab7 (37) showing a collapse of the endosomal system typified by swollen endosomes positive for both endosomal and lysosomal markers. To test whether the defective trafficking of cathepsin D in LRRK2-KO cells is also coupled with defective endo-lysosomal organization we quantified colocalization between cathepsin D and the early endosomal localized CI-M6PR (Fig. 7G and H). As expected in wild-type cells the two proteins showed partial colocalization onto small (500 nm to 1 μm) punctate endosomes. However, in LRRK2-KO cells we observed a significant increase in the colocalization between the two markers indicating a fundamental defect in the organization of the endo-lysosomal system. Therefore, in both kidney cells and tissue we observed an increase in colocalization between cathepsin D and the CI-M6PR suggesting impaired receptor recycling in the absence of LRRK2. These results show that deficiencies in the organization of the endo-lysosomal system represent a primary event in response to loss of LRRK2.

## Discussion

Understanding the physiological function of LRRK2 has implications for nominating biological processes relevant to PD and may be important for predicting safety and tolerability of LRRK2 kinase inhibitors if these compounds proceed to clinical trials. Here, we show that genetic ablation of LRRK2 is associated with altered levels of proteins related to lysosomal, cytoskeletal and protein translation pathways *in vivo*. In cells, we find enhanced trafficking of the CI-M6PR to lysosomes, potentially identifying a very early consequence of LRRK2 inhibition resulting in the observed accumulation of lysosomal proteases over time. In contrast, mice harboring the G2019S allele do not display any overt phenotypic difference from control mice and show only minimal changes in protein expression compared with the clear changes in LRRK2-KO mouse kidneys. Our observations showing no clear effects on protein abundance argue against a simple dominant negative mechanism for the G2019S mutation in PD kidneys and/or brain.

Prior studies of LRRK2-KO mice have identified multiple abnormalities in kidneys (30,32,38). Here, we have extended past approaches to show more defined alterations in multiple lysosomal proteases, cytoskeletal proteins and protein translational components. Changes in the latter two protein categories are consistent with the reported biology of LRRK2. For example, LRRK2 was shown to bind tubulin directly (21), influence actin dynamics (39) and may regulate phosphorylation of the microtubule binding protein tau (40–45). Similarly, overexpression of proteins that regulate translation, including 4EBP1 (25,46) and RPS15 (47), can mitigate toxic effects of mutant LRRK2. Overall, the broad range of proteins that are differentially regulated in our proteomics analysis suggests that endogenous LRRK2 affects multiple proteins in three major categories.

This study suggests that loss of *Lrrk2* influences lysosomal, cytoskeletal and protein translation and that defective intracellular protein trafficking is the primary cause. We were able to show that localization of CI-M6PR is altered in LRRK2-KO cells, suggesting inherent alterations in trafficking in these cells. Interestingly, we find greater colocalization between CI-M6PR and cathepsin D. This could indicate either that cells are increasing the rate of trafficking to the lysosome or are failing to recycle CI-M6PR after delivery. As LRRK2 phosphorylates multiple members of the Rab family of small GTPases (48–50) one potential testable hypothesis that emerges from these observations is that KO cells and tissues upregulate protein trafficking pathways to compensate for loss of *Lrrk2*-dependent Rab phosphorylation (51,52). It is also possible that the role of LRRK2 in the membrane trafficking pathways here studied is not mediated simply by its kinase activity but by other functional protein interaction domains such as the GTPase Roc-COR domain which seems involved in retrograde trafficking via downstream Rab GTPases such as Rab29 (53). In addition, LRRK2 could play a scaffolding role along microtubules, helping the formation of signaling complexes involved in membrane trafficking (21).

The distinction between specific regulation of individual proteins and broad alterations in categories of biologically related molecules is important as it suggests some of the observed changes may be compensatory responses to loss of *Lrrk2*. Supporting this interpretation, our data shows that differences between genotypes increase slowly over several months then tend to diminish partially with age, consistent with prior data suggesting complex biphasic responses with aging in LRRK2-KO animals (18). We therefore propose that *Lrrk2* is involved in multiple cellular pathways and that, to compensate for lack of *Lrrk2*, there are compensatory and co-ordinated responses in the same pathways.

The relevance of this detailed study in brain and kidneys in loss of function and gain of kinase function models lies first in the identification of novel categories of proteins affected by loss of *Lrrk2*. These findings lay emphasis on the concept that kinase inhibitors might affect several intracellular pathways accumulatively over time. Second, the lack of concordance in proteomic changes between LRRK2-KO and the G2019S knock-in animals supports prior data that the mutations in *Lrrk2* are pathogenic owing to gain of function rather than having dominant negative effects.

Overall, the current data shed light on multiple protein networks affected by loss of LRRK2 in early stages of mouse development and demonstrates the utility of unbiased approaches to explore *in vivo* effects of gene manipulation.

## Materials and Methods

### Animals

Experiments using animals were conducted in compliance with the Guide for the Care and Use of Laboratory Animals of the National Institutes of Health. The specific experiments performed in this study were approved by the Institutional Animal Care and Use Committees of the US National Institute on Aging (Animal study protocol number 463-LNG-2018). Animal experiments performed at UCL were approved by the UCL Ethics Committee and by the home office as detailed in the relevant project license 80/2486 (KH).

LRRK2-KO mice (54) were generously provided by Huaibin Cai, NIA. LRRK2 G2019S knock-in mice were generated as described previously (55). For proteomics experiments and for validation by western blotting, age-matched WT, homozygous LRRK2-KO mice and heterozygous knock-in mice, each bred on a C57BL/6J background, were euthanized by cervical dislocation. For additional biological validation of the effects of age on protein expression, we generated separate cohorts of WT and LRRK2-KO mice. From each animal, one kidney was immediately frozen in dry ice while the other was fixed in 4% PFA in PBS for histological analysis. Brain hemispheres were separated and cortex, striatum and hippocampus were dissected and frozen in dry ice.

### Kidney sample preparation

For iTRAQ proteomic experiments, frozen kidneys dissected from 1-year-old mice were homogenized using Dounce homogenizers in isolation buffer containing 0.225 M mannitol, 0.05 M sucrose, 0.0005 M HEPES, 1 mM EDTA. The homogenate was centrifuged at 2000×*g* for 3 min at 4°C and the supernatant then collected in a separate tube and centrifuged at 12 000×*g* for 8 min at 4°C. The supernatant was then centrifuged at 100 000×*g* for 30 min at 4°C and the resulting 10K supernatant and pellet fractions retained. The 10K pellets were resuspended in iTRAQ compatible buffer (0.3 M HEPES, 2% CHAPS, 1 mM EDTA). Total kidney homogenates for immunoblot validation of additional cohorts were prepared in lysis buffer (20 mM Tris–HCl, 1% NP40, 150 mM NaCl, 1 mM EDTA, 10% glycerol), with protease inhibitor cocktail tablet (Roche) and Halt phosphatase inhibitor (ThermoScientific) added immediately before use. Protein concentrations were determined using 660 nm protein assay reagent (Pierce) using bovine serum albumin as a standard reference.

### Primary kidney cells preparation

For primary kidney cell preparation, kidneys from 8- to 9-month-old mice were collected and washed in cold HBSS (Hank Balances Salt Solution, Gibco) to remove residual blood. Renal fibrous capsule, connective tissue and renal medulla were removed. Kidney cortical tissue was dissected and mechanically minced using scalpels. Tissue homogenates were then transferred into 50 ml falcon tubes containing HBSS with 1 mg/ml Collagenase IV (Invitrogen) to allow digestion at 37°C for 30 min. Digested kidney fragments were then passed through a 100 μm sieve in another falcon tube to remove fibrous tissue. The sieve was washed in HBSS and the cell suspension centrifuged at 1200 rpm for 5 min two times. Finally, the cell pellet was resuspended in complete culture media: DMEM/F12 1:1 media (Invitrogen) containing 5% FBS, 10 ng/ml epidermal growth factor (Invitrogen), 1% penicillin/streptomycin (Invitrogen), 1% l-glutamine (Invitrogen), 50 mM hydrocortisone (Sigma Aldrich), 5 μg/ml insulin (Invitrogen), 5 μg/ml transferrin (Sigma Aldrich) and 50 nM sodium selenite (Sigma Aldrich). Cells were cultured in 75 cm^2^ plastic flasks with complete culture medium at 37°C under 5% CO_2_ in a humidified atmosphere.

### Isobaric tag for relative and absolute quantitation (iTRAQ) labeling

For iTRAQ experiments, we modified procedures we previously reported for mouse brain samples (56). Kidney 10K supernatants and pellets from five WT and five LRRK2-KO samples were analyzed in two separate experiments for a total of four iTRAQ runs (Fig. 1). A pooled reference sample was made by combining 40 μg of each of the WT samples together. The concentration of each sample was adjusted to 1 μg/μl with iTRAQ buffer (0.3 M HEPES, 2% CHAPS, 1 mM EDTA). Samples were treated with 2 μl of tris(2-carboxyethyl)phosphine for 60 min at 60°C and subsequently alkylated with 1 μl of methyl methanethiosulfonate for 20 min at RT. The mass spectrometry grade trypsin (Promega) was used to digest the samples. The first digestion was carried out overnight at 37°C with a trypsin:protein ratio of 1:100 (w/w). The samples were digested again with a trypsin:protein ratio of 1:100 (w/w) for 8 h at 37°C. Before addition to samples, iTRAQ eight-plex reagents were dissolved in 150 μl of isopropanol and vortexed. Each sample mixture was then labeled for 3 h at RT with the appropriate iTRAQ reagent, and labeled peptide mixtures were combined. Samples were desalted using an Oasis HLB 200 mg cartridge. Fractionation of iTRAQ labeled peptides by liquid chromatography (LC) and subsequent tandem mass spectrometry (MS/MS) analyses of the fractions were performed as previously described (56).

### Western blotting

Equal amounts of total protein from each preparation were separated using SDS-PAGE and transferred into nitrocellulose membranes. After 1 h blocking with Odyssey Blocking Buffer (LI-COR), membranes were probed overnight with primary antibodies (listed in the following section). Incubation with IRDye-labeled 800CW goat anti-rabbit and goat anti-mouse secondary antibodies (LI-COR, 1:15 000) was performed for 1 h at RT. Protein bands of interest were visualized with Odyssey CLx Infrared Imaging Studio. Band intensity was quantified using LI-COR Image Studio software.

### Antibodies

The following antibodies used for immunoblot were diluted in a 1:1 mix of Odyssey Blocking Buffer (LI-COR) and TBS containing 0.1% (v/v) Tween 20:rabbit polyclonal antibody to cathepsin D (Millipore, 219361, 1:1000), goat polyclonal antibody to cathepsin D C-20 (Santa Cruz, sc-6486, 1:200), rabbit polyclonal antibody to legumain (Abcam, ab125286, 1:1000), mouse monoclonal antibody to gephyrin (Synaptic System, 147 111, 1:2000), mouse monoclonal antibody to β-actin (Sigma, A5316, 1:10 000), rabbit polyclonal antibody to α/β-tubulin (Cell Signaling, 2148, 1:1000), mouse monoclonal antibody to acetylated-tubulin (Sigma, T7451, 1:1000), rabbit polyclonal antibody to coronin 1C (Proteintech, 14749-1-AP, 1:1000), mouse monoclonal antibody to NAA15 (LSBio, LS-C342562, 1:1000), rabbit polyclonal to EEF1G (Abcam, ab72368, 1:1000), mouse monoclonal to HNRNPK antibody (Abcam, ab23644, 1:1000) and rabbit polyclonal to LC3B (Abcam, ab51520, 1:2000). The following antibodies were used for immunohistochemistry: rabbit polyclonal antibody to cathepsin D (Millipore, 219361, 1:200), goat polyclonal antibody to cathepsin D C-20 (Santa Cruz, sc-6486, 1:200), rat monoclonal antibody to LAMP1 (1D4B) (Abcam, ab25245, 1:200), mouse monoclonal to mannose-6-phosphate receptor cation independent (CI-M6PR) antibody [2G11] (Abcam, ab2733, 1:200), rabbit monoclonal to CI-M6PR [EPR6599] (Abcam, ab124767, 1:200).

### Histological analysis

Kidneys were fixed *post-mortem* by immersion in 4% PFA overnight and then transferred to PBS with 30% sucrose and 0.05% sodium azide for 24 h. Tissues were then frozen and 40 μm sections cut using a Leica CM1900 cryostat. After three 5 min washes in PBS, sections were blocked at room temperature for 1 h in blocking buffer (0.3% Triton, 1% BSA, 1% donkey serum in PBS). The same buffer was used for the primary antibody incubation overnight at 4°C with gentle shaking. Sections were then washed 3 times in PBS for 10 min and incubated with Alexa Fluor secondary antibodies (donkey anti-rabbit and donkey anti-goat, 1:500) for 2 h at room temperature. After three 10 min washes in PBS, sections were mounted on glass slides using Prolong Gold mounting media (Life Technologies).

### Immunocytochemistry

Primary kidney cells were seeded in poly-d-lysine coated coverslips and fixed in 4% PFA for 20 min. Cells were then incubated for 1 h with blocking buffer (0.1% Triton, 5% FBS in PBS) and the same buffer was used for the following antibody incubations. Primary antibody incubation was performed for 2 h at RT. Cells were then washed three times in PBS prior to secondary antibody incubation for 1 h with Alexa Fluor antibodies diluted 1:500. Each coverslip was then mounted on a microscope slide using Prolong mounting media (Sigma) and left to dry overnight at RT.

### Confocal microscopy

Confocal microscopy was performed using a Zeiss LSM 710 (Carl Zeiss). Fluorescence was excited by the 405, 488 and 595 nm laser lines of Argon, diode and Helium/Neon lasers, respectively. Images were taken with a 40× 1.3 numerical aperture (NA) or 63×1.4 NA oil objective. Images were collected using Zen software (Carl Zeiss) and prepared using Fiji (NIH). Representative images are shown without any additional processing. Airyscan imaging was performed using a Zeiss 880 outfitted with an Airyscan module. Data were collected using a 63×1.4 NA objective and immersion oil optimized for 30°C (Carl Zeiss). Colors were collected sequentially to minimize crosstalk, and Airyscan processing was performed using the Airyscan module in the commercial ZEN software package (Carl Zeiss).

### High content imaging

Cells that had been fixed and immunostained for confocal analysis were subsequently analyzed using a ThermoScientific Cellomics ArrayScan VTI HCS Reader. Plates were imaged using a 20× objective and analyzed using the Spot Detector bioapplication for percent of cells with cathepsin D-positive spots from total number of cells, average spot area and intensity of staining per cell.

### Image quantification

Quantification was performed by an individual blinded to genotype using a combination of custom algorithms in Fiji (NIH), Imaris (Biplane, Inc.) and in NIS Elements analysis software. To assist the algorithm in correctly identifying the structures, background subtraction was performed by subtracting a flat numerical value from all channels in all images. The number of cathepsin D-positive vesicles was quantified using a spot detection algorithm in Imaris, thresholds were decided from several images and then applied to all images simultaneously. To normalize the number of cathepsin D-positive vesicles to the number of cells in the tissue section, the nuclei were quantified using a surface detection algorithm using the method of marching squares.

### Quantitative real-time polymerase chain reaction

Isolation of total RNA, reverse transcription and quantitative real-time polymerase chain reaction (qRT-PCR) were performed as previously described with few changes (57). Total RNA from primary cultured mouse kidney cells and mouse kidney tissue was isolated and purified with the RNeasy Mini Kit (QIAGEN). RNA was reverse transcribed with the SuperScript^®^ III First-Strand Synthesis System (Invitrogen) and qPCR of diluted cDNA was carried out on an AriaMx Realtime PCR System (Agilent Technologies) using iTaq™ Universal SYBR^®^ Green Supermix (Bio-Rad Laboratories) for a total reaction volume of 20 µl. Oligo 6.0 software (MedProbe) served as program to select intron-spanning primers targeting the *mus musculus* cathepsin D *g*ene (NM_009983.2; 330-forward 5′-GCCGCAGTGTTTCACAG-3′; 479-reverse 5′-TGAGCCGTAGTGGATGTCAA-3′; amplification product: 169 bp). Optimized primers for the two housekeeping reference genes, GAPDH and β-actin, have been used as published earlier (57): GAPDH: NM_008084; 205-forward 5′-GCAAATTCAACGGCACA-3′; 337-reverse 5′-CACCAGTAGACTCCACGAC-3′; amplification product: 141 bp; β-actin: NM_007393; forward 5′-GCCAACCGTGAAAAGATGAC-3′; reverse 5′-GGCGTGAGGGAGAGCATAG-3′. In order to verify the specificity of the PCR products gel electrophoresis, melting curve analysis and ‘-RT’ (reverse transcriptase) as well as water control PCR reactions were conducted. Calculation of relative expression levels for cathepsin D in LRRK2 KO versus WT samples was determined with the ΔΔC_t_ method. Statistical analysis was done using non-parametric Mann–Whitney test.

### Bioinformatics and statistical analyses

In all experiments, *n* represents the number of individual animals included in an experiment. Both male and female mice were used. Although animals were not treated and therefore not randomized into treatment groups, iTRAQ proteomics experiments were performed with animals of both genotypes included across different proteomics runs (Fig. 1A). In all statistical analyses, a preset value of *α* = 0.05 was used to reject the null hypothesis of no difference, with multiple test correction as appropriate.

iTRAQ labeled peptide identification and quantitation were performed using Mascot (58) from Xcalibur RAW files with parameters and thresholds for peak picking as previously described (56). Peptide identification was performed using the Mascot Server (version 2.5) to identify homologous peptides in the Sprot Mouse Database (Uniprot Proteome ID: UP000000589) with iTRAQ8plex (N-term) and iTRAQ8plex (K) set as fixed modifications and Methylthio (C), Oxidation (M) and iTRAQ8plex (Y) set as variable modifications. Only unique peptides were used to identify and calculate protein ratios. The iTRAQ label intensity for each sample was divided by the intensity of the pooled reference to obtain peptide ratios, and subsequently normalized so that the median ratio for each peptide was 1 for the WT animals. Missing data points were imputed using *k*-nearest neighbors in the ‘impute’ package in R within each sample type and series only where the number of missing values was <5 (59). Statistical inferences were assessed by Welch’s *t*-test, allowing for unequal variance between groups, followed by the Benjamini–Hochberg false-discovery rate correction for multiple testing (60). Graphs are plotted as raw *P* values, but reported as significant only if the adjusted *P* < 0.05. Evaluation of Gene Ontology term enrichments was performed using gProfileR within R (61). Protein interaction networks were acquired from the IntAct database (http://www.ebi.ac.uk/intact/; date last accessed July, 2016) and visualized using Cytoscape.

Data were plotted using Prism 6 (Graphpad) or R (https://www.rstudio.com/; date last accessed July, 2016) and displayed as means and standard error of the mean. For validation experiments, Student’s *t*-test was used to determine differences between data consisting of two groups and ANOVA with Bonferroni post hoc tests for multiple group comparisons. Differences between groups were plotted on graphs using the following codes: *P < 0.05, **P < 0.01, ***P < 0.001, ****P < 0.0001.

## Supplementary Material

Supplementary Data
