# Supplementary material for: Proteomic analysis reveals co-ordinated alterations in protein synthesis and degradation pathways in LRRK2 knockout mice
Source: Hum Mol Genet. 2018 Jun 18;27(18):3257–71. doi: 10.1093/hmg/ddy232 (PMC6121185; doi:10.1093/hmg/ddy232)

Figure S1. Categorization of proteins showing altered abundance in LRRK2 knockout kidneys

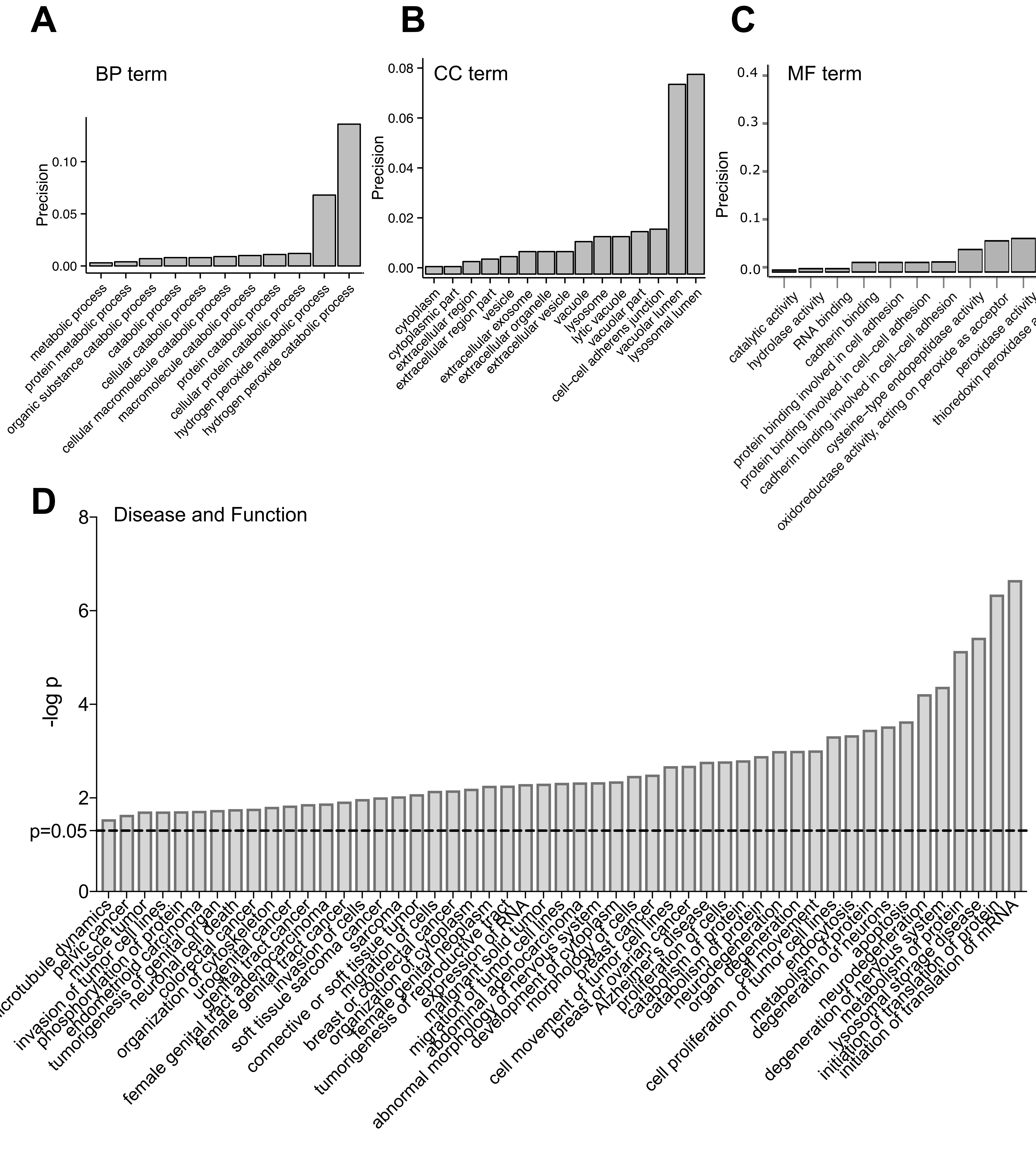

Figure S2. Independent iTRAQ experiments in LRRK2 knockout kidneys

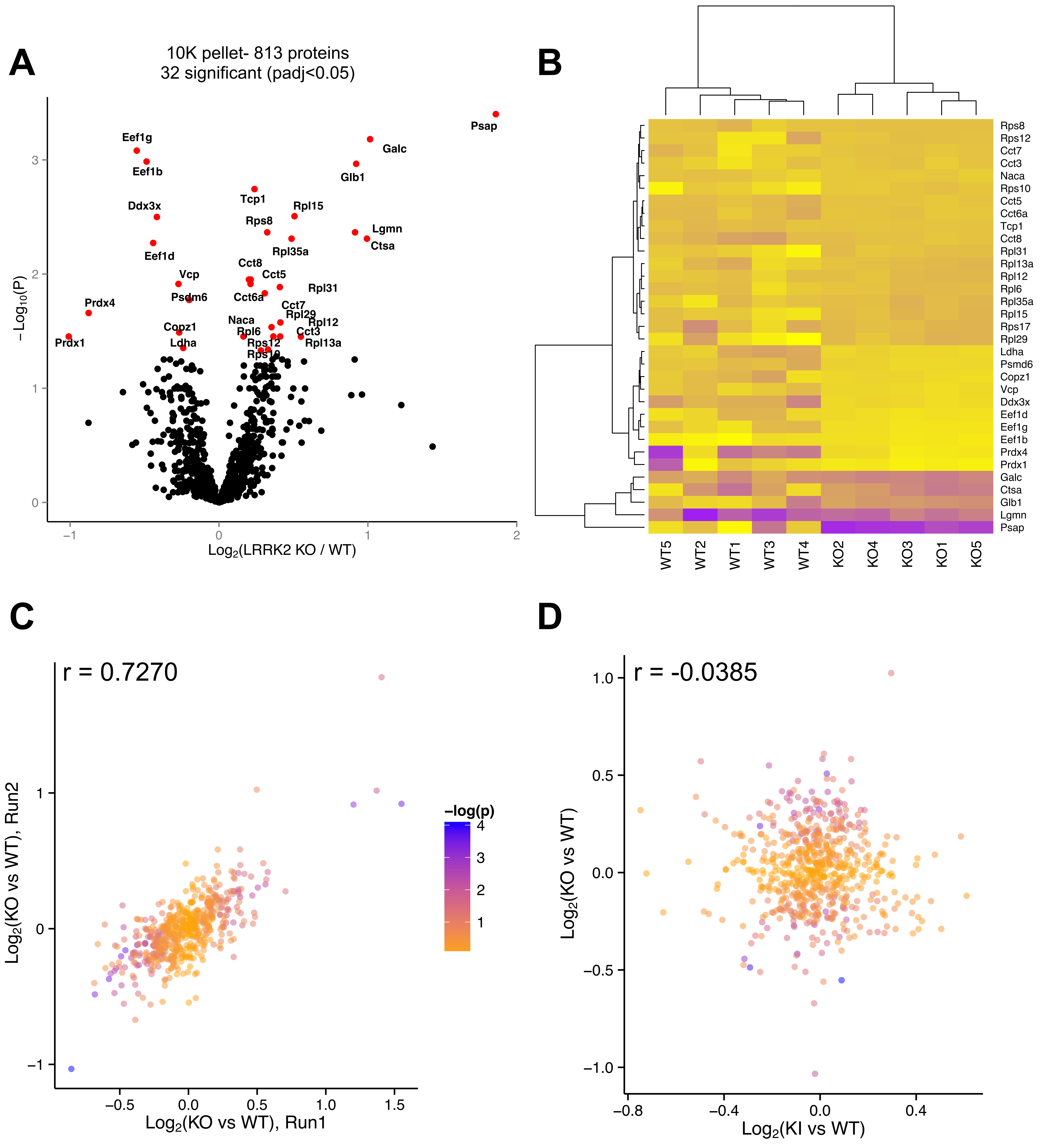

Figure S3. No differences in proteomics candidates in LRRK2 knockout brains

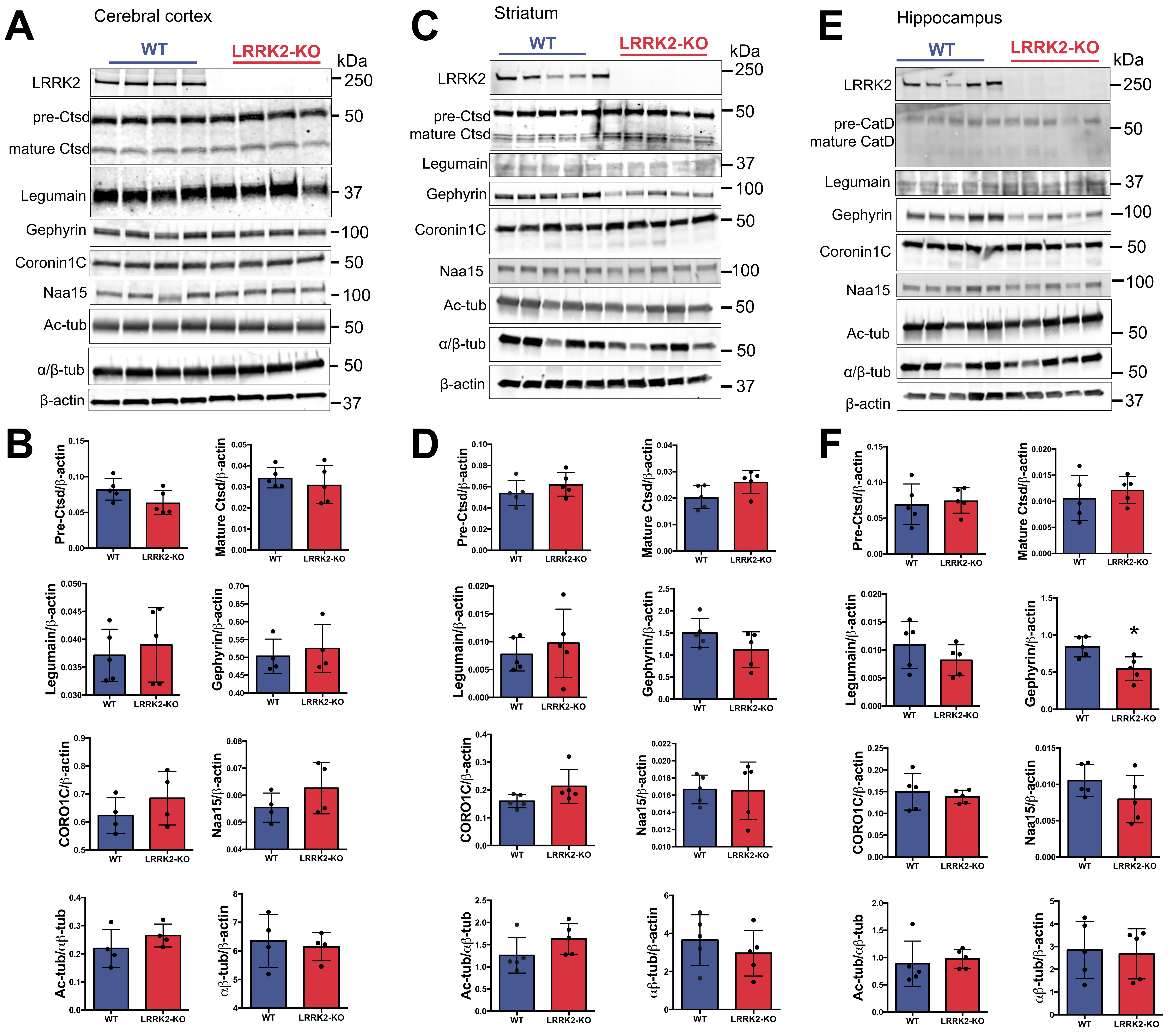

Figure S4. Additional quantifications of immunoblots from aging cohorts

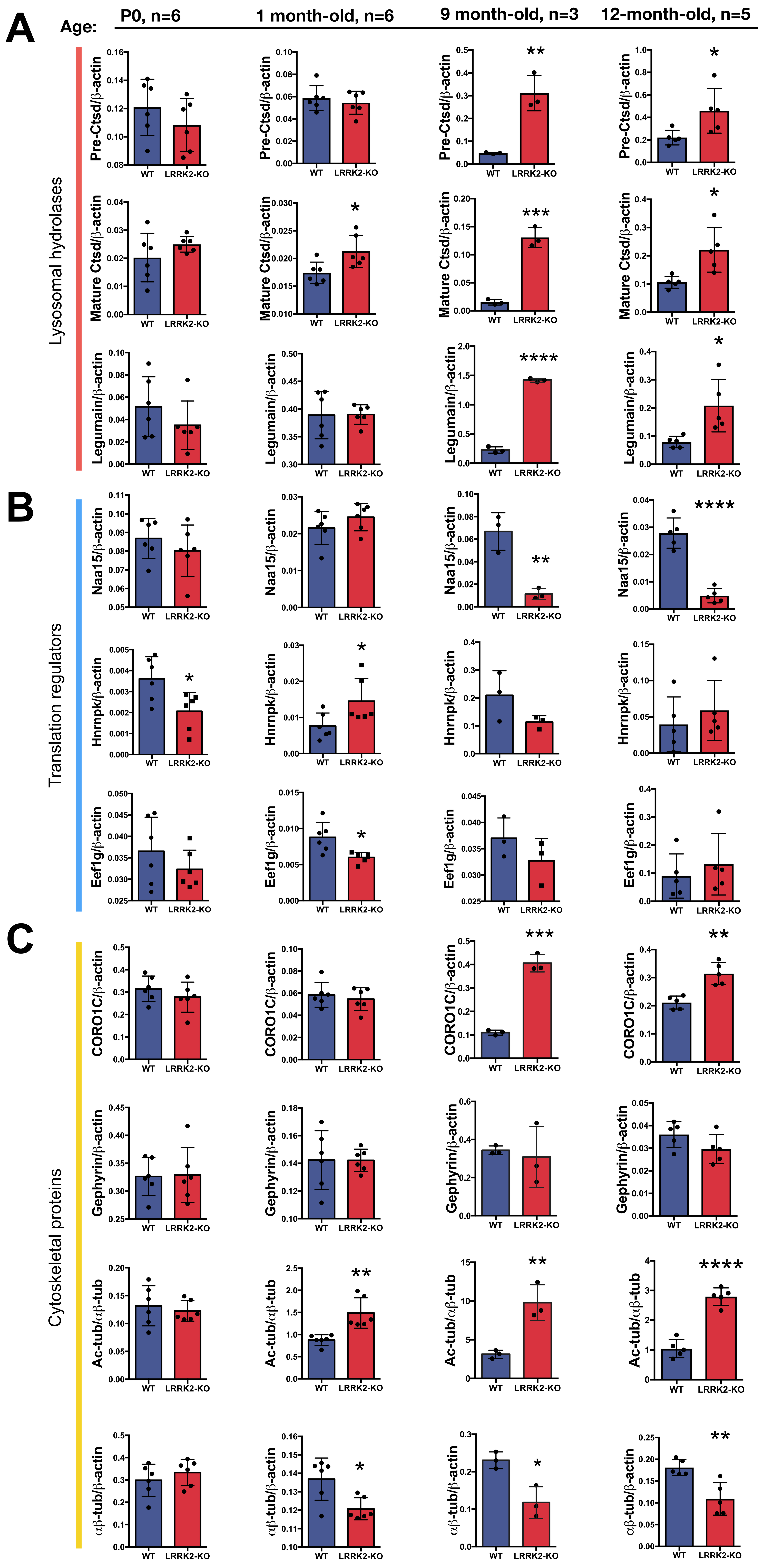

Figure S5. LRRK2 knockout kidneys enlarged size and morphological alterations

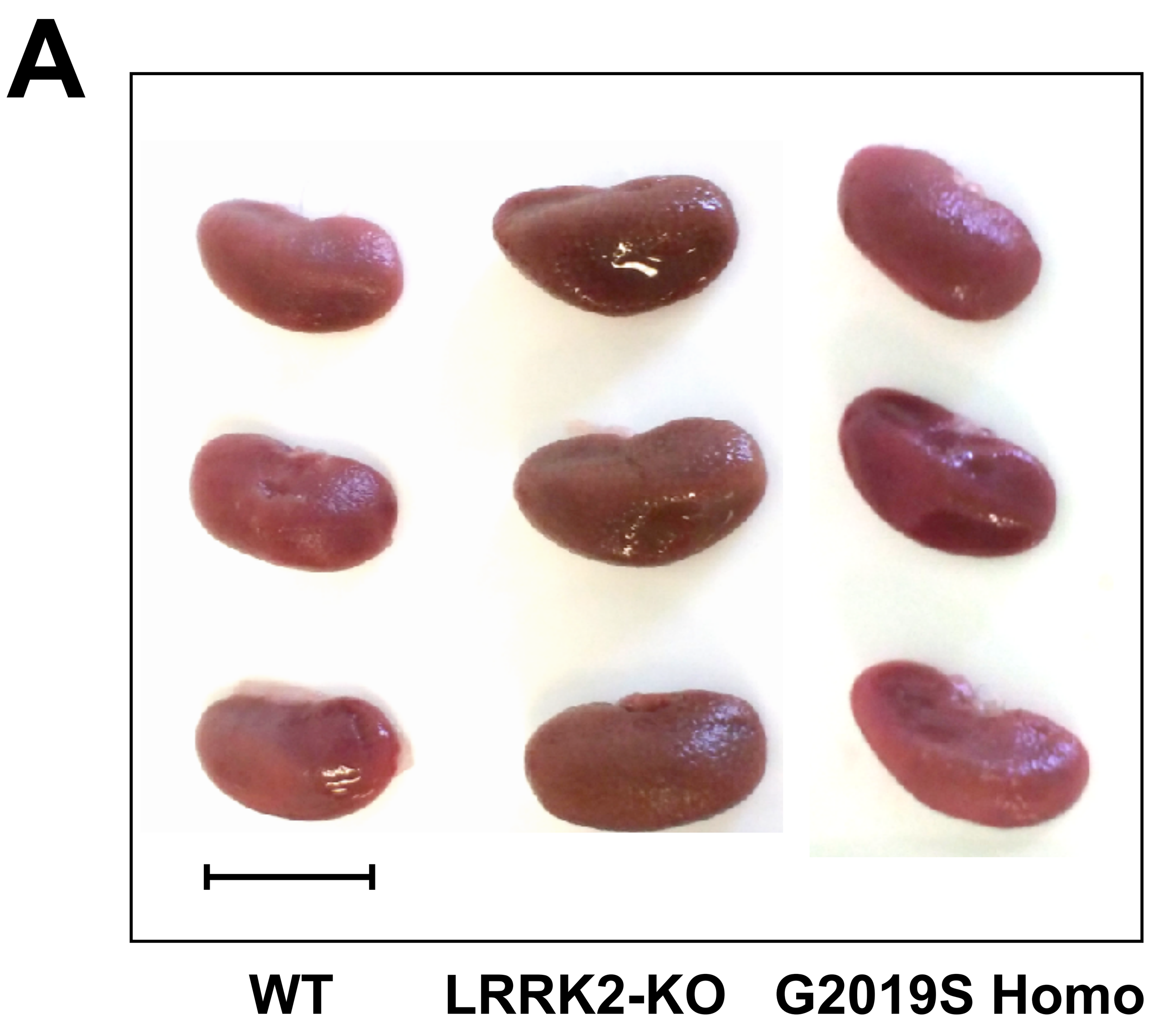

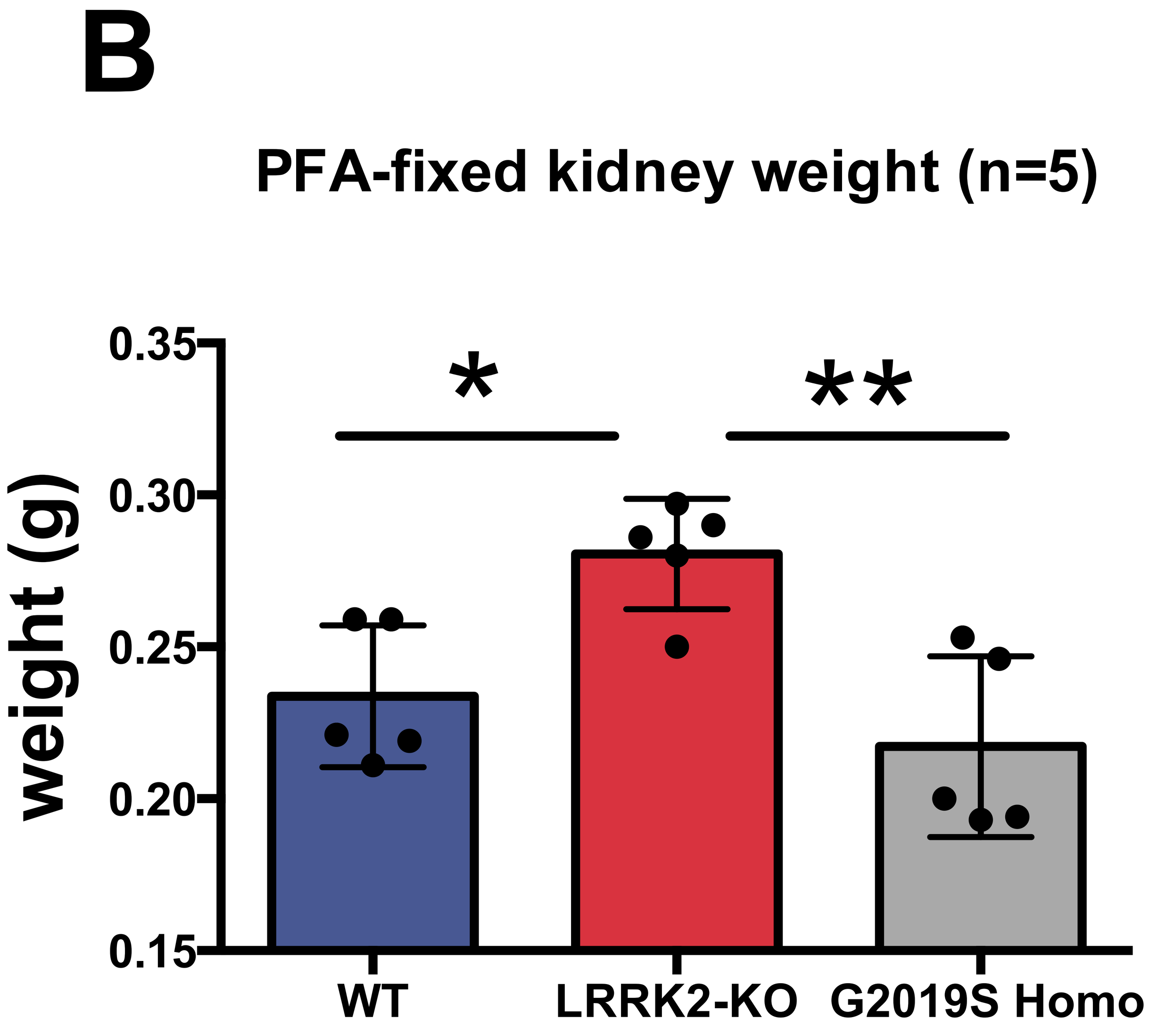

Figure S6. Loss of LRRK2 does not affect cathepsin D transcription and translation

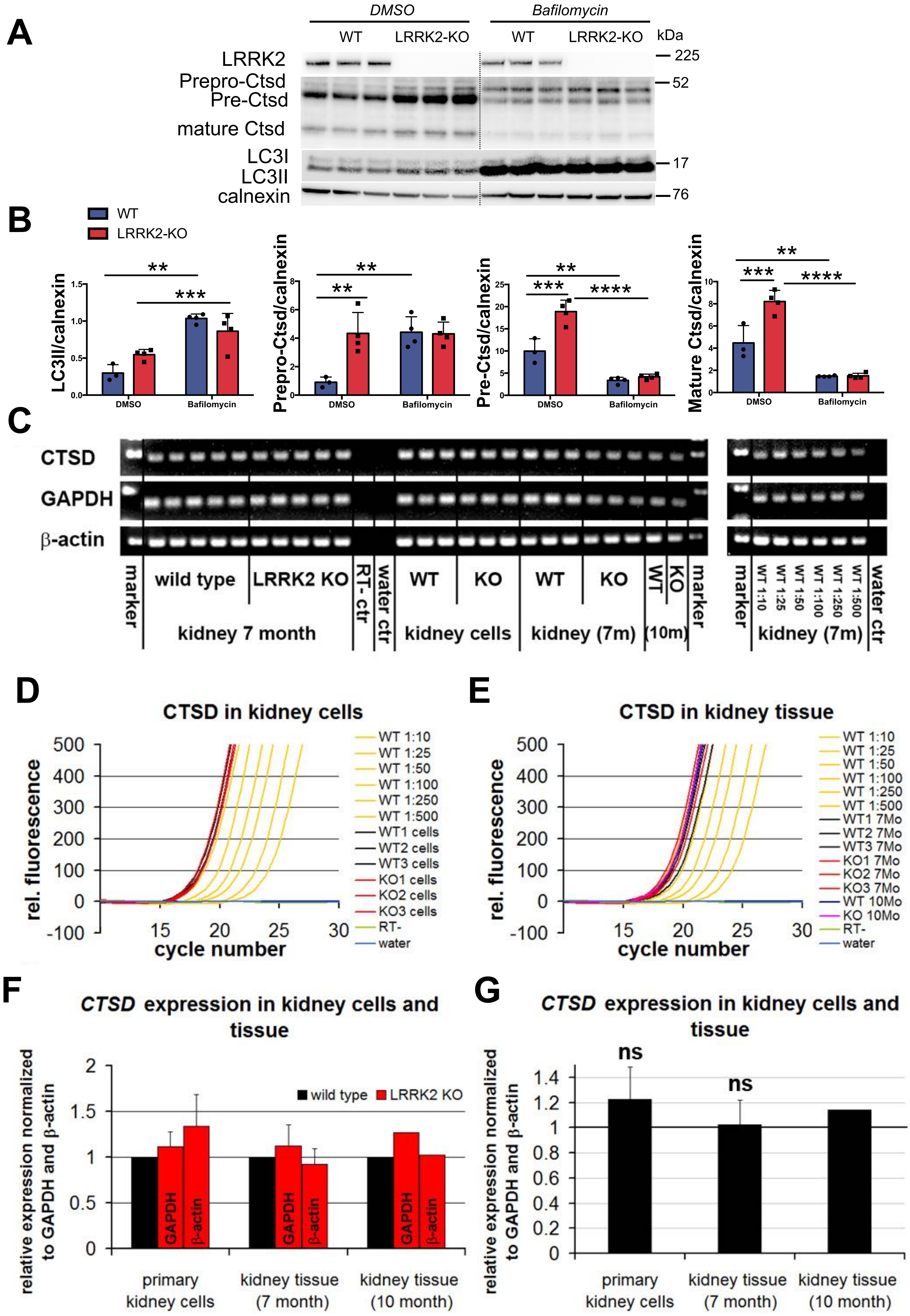

Figure S7. Cathepsin D localisation to lysosomal compartments is altered in LRRK2-KO kidney cells

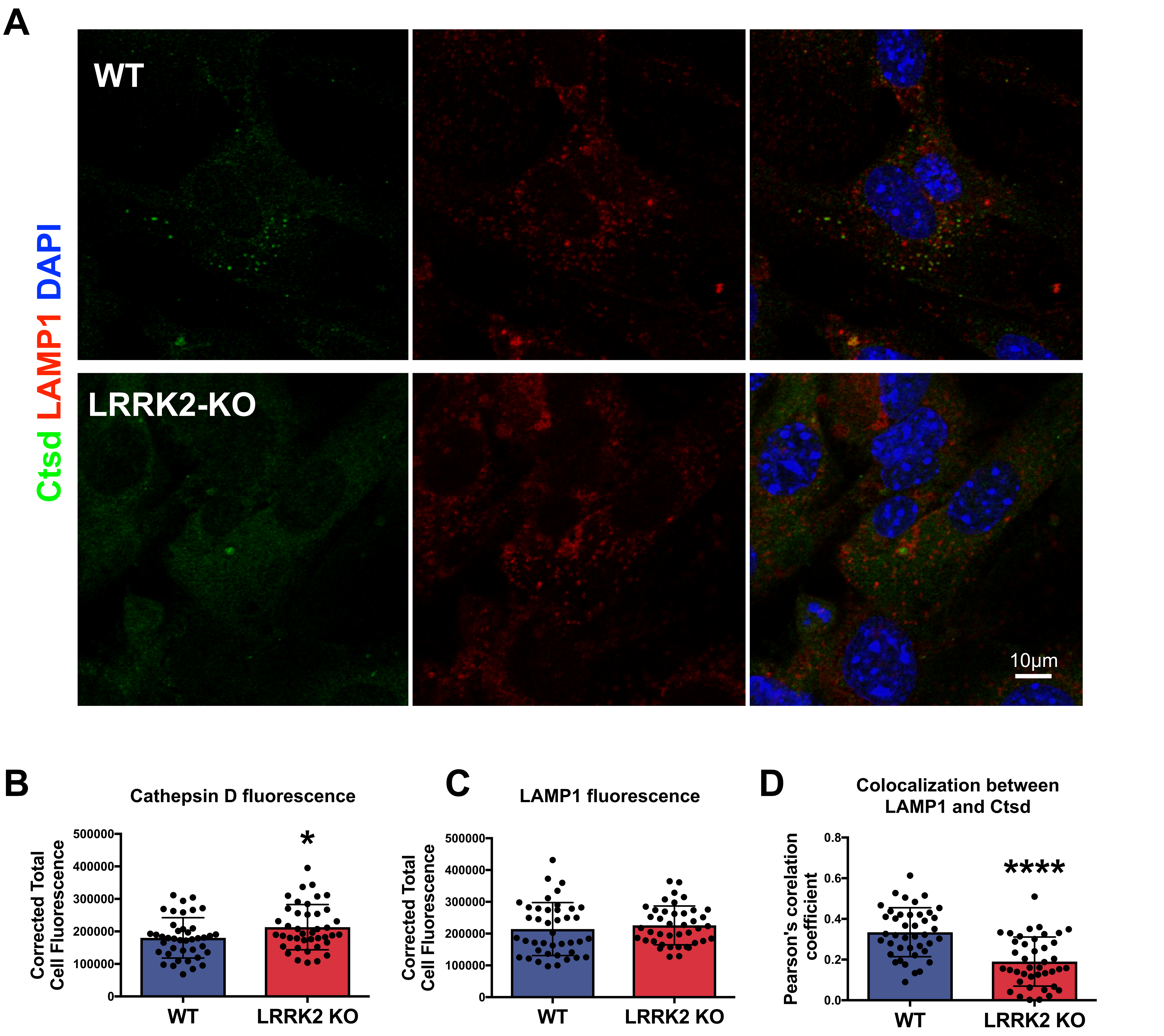

Uncropped immunoblots from 10K supernatants (12-month-old)

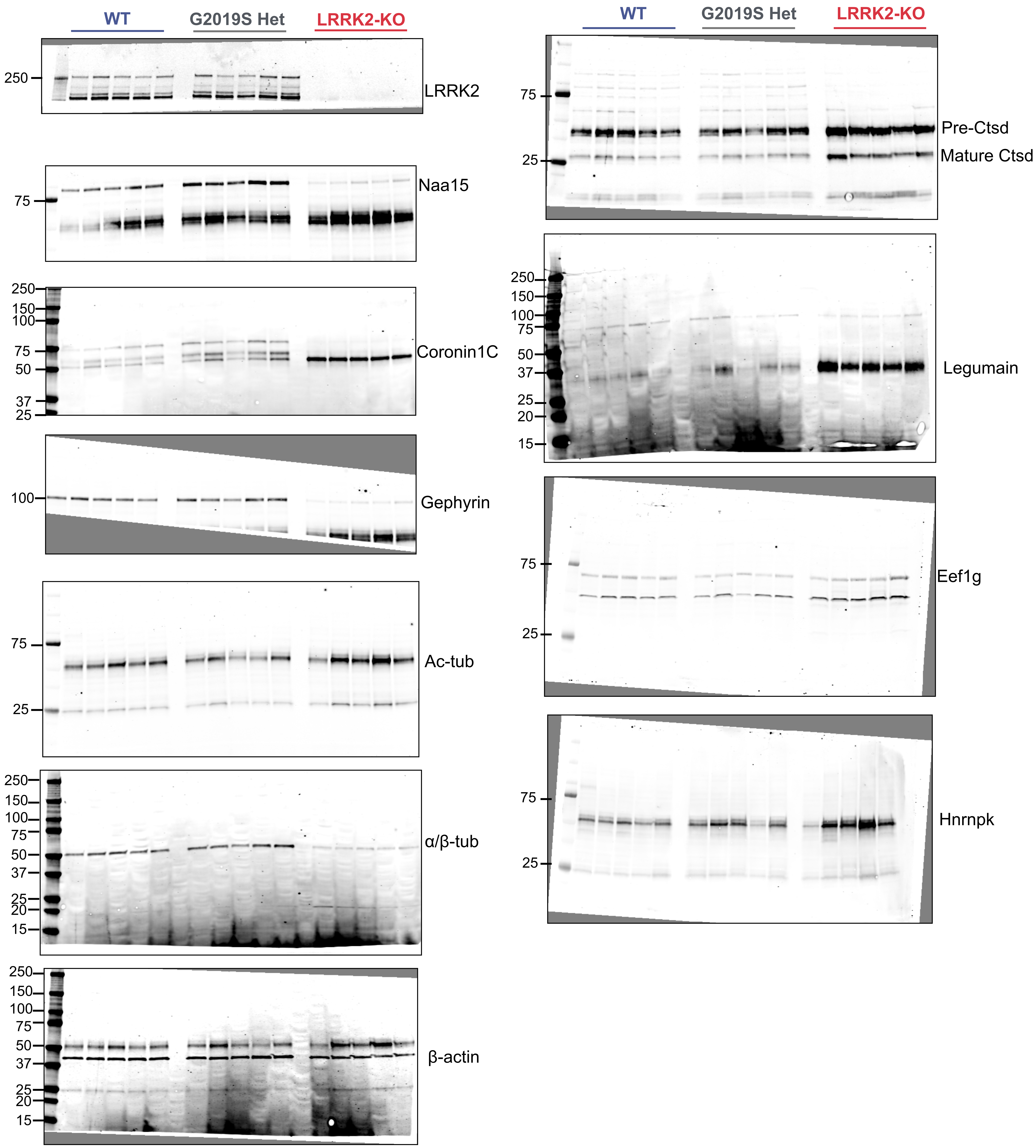

Uncropped immunoblots from 10K pellets (12-month-old)

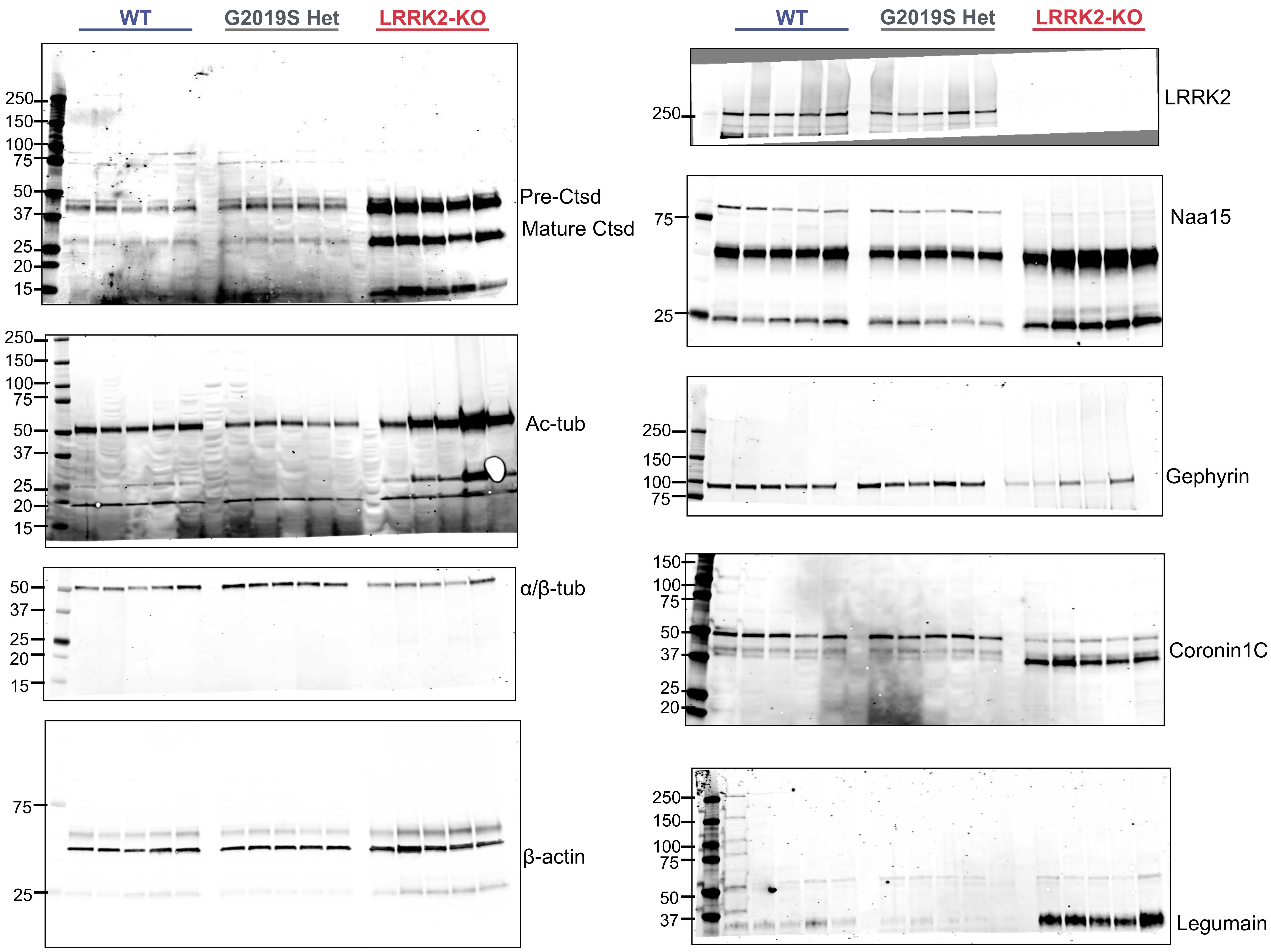

Uncropped immunoblots from kidney homogenates (P0, n=6)

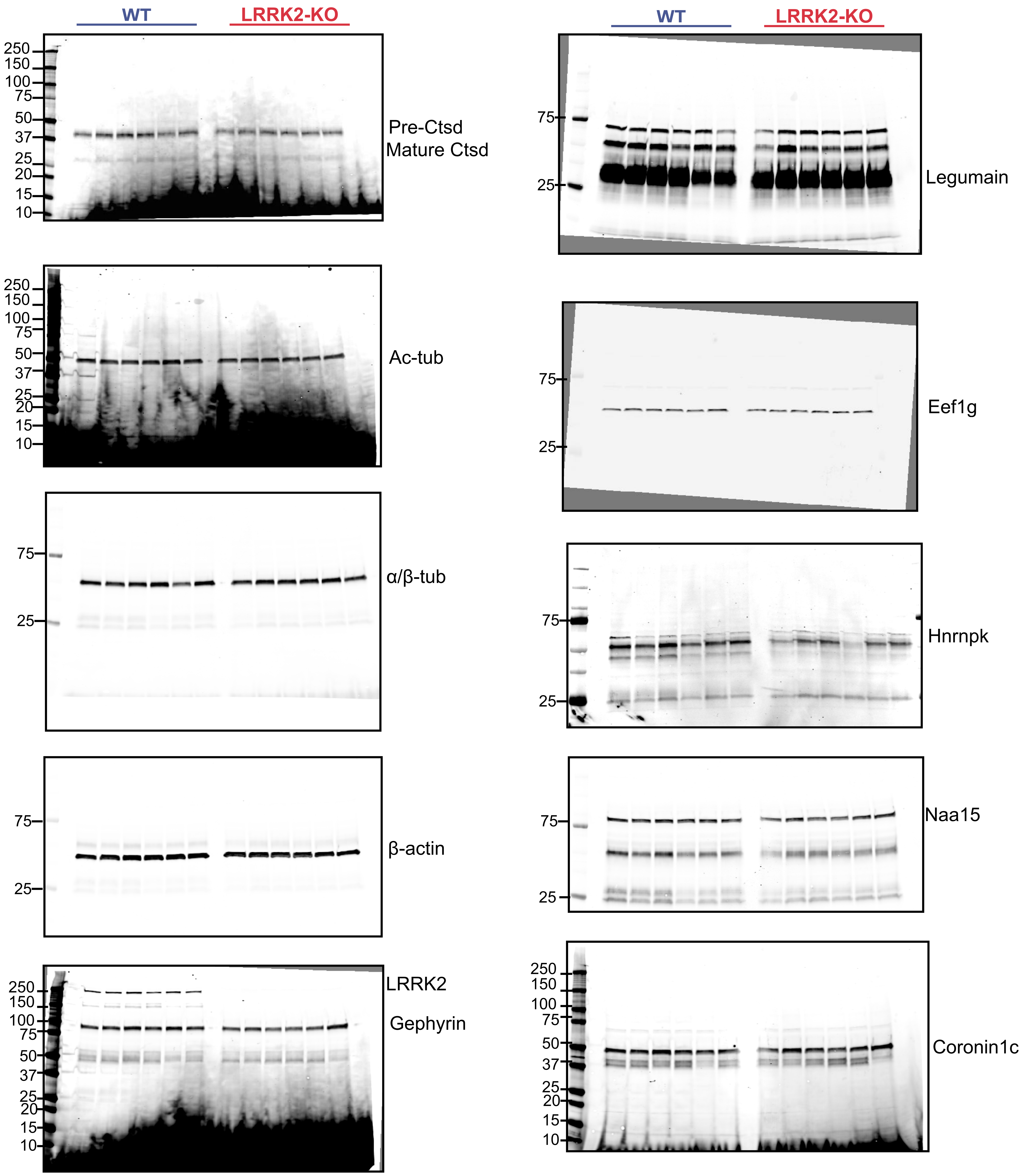

Uncropped immunoblots from kidney homogenates (1 month-old, n=6)

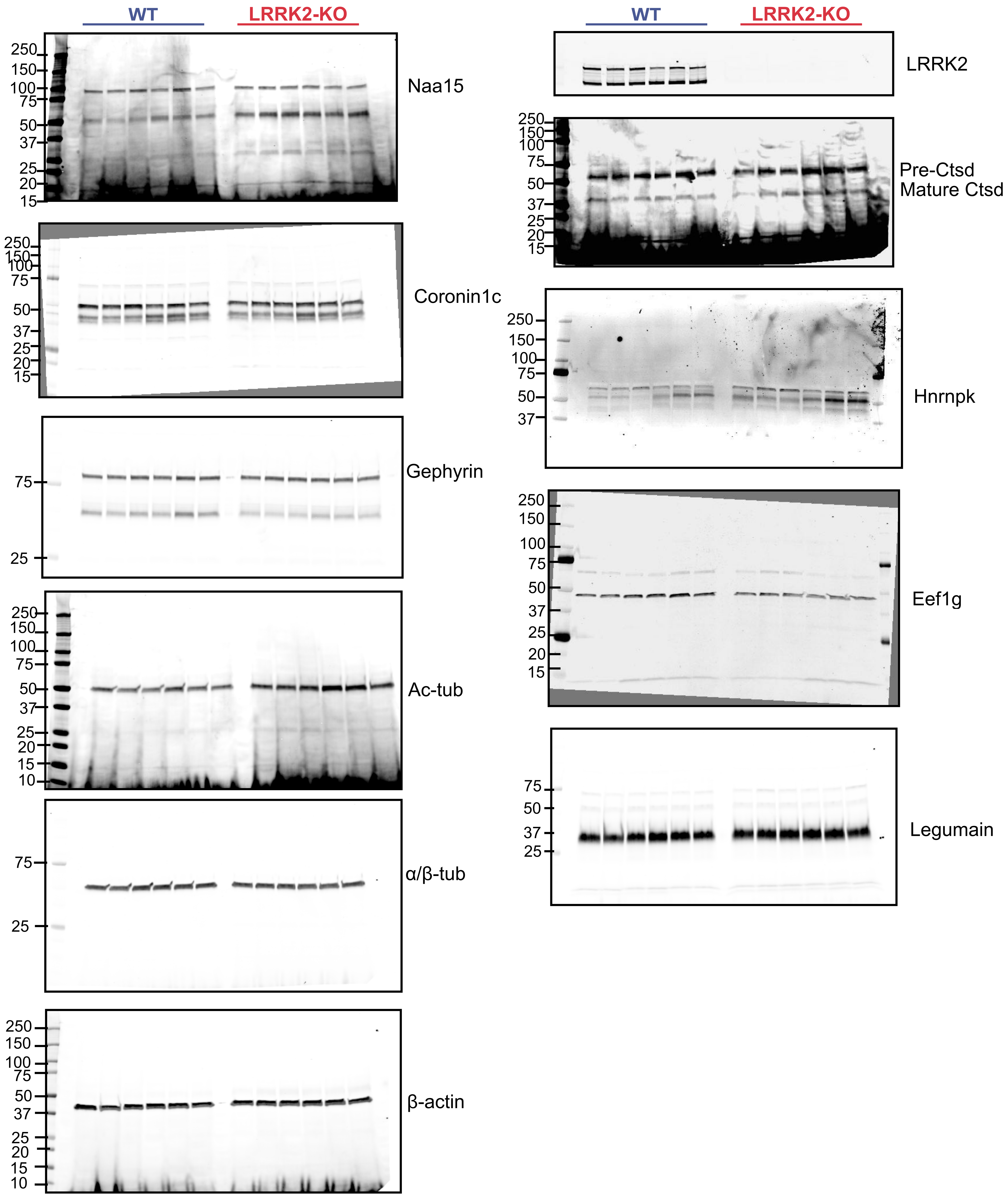

Uncropped immunoblots from kidney homogenates (9 month-old, n=3)

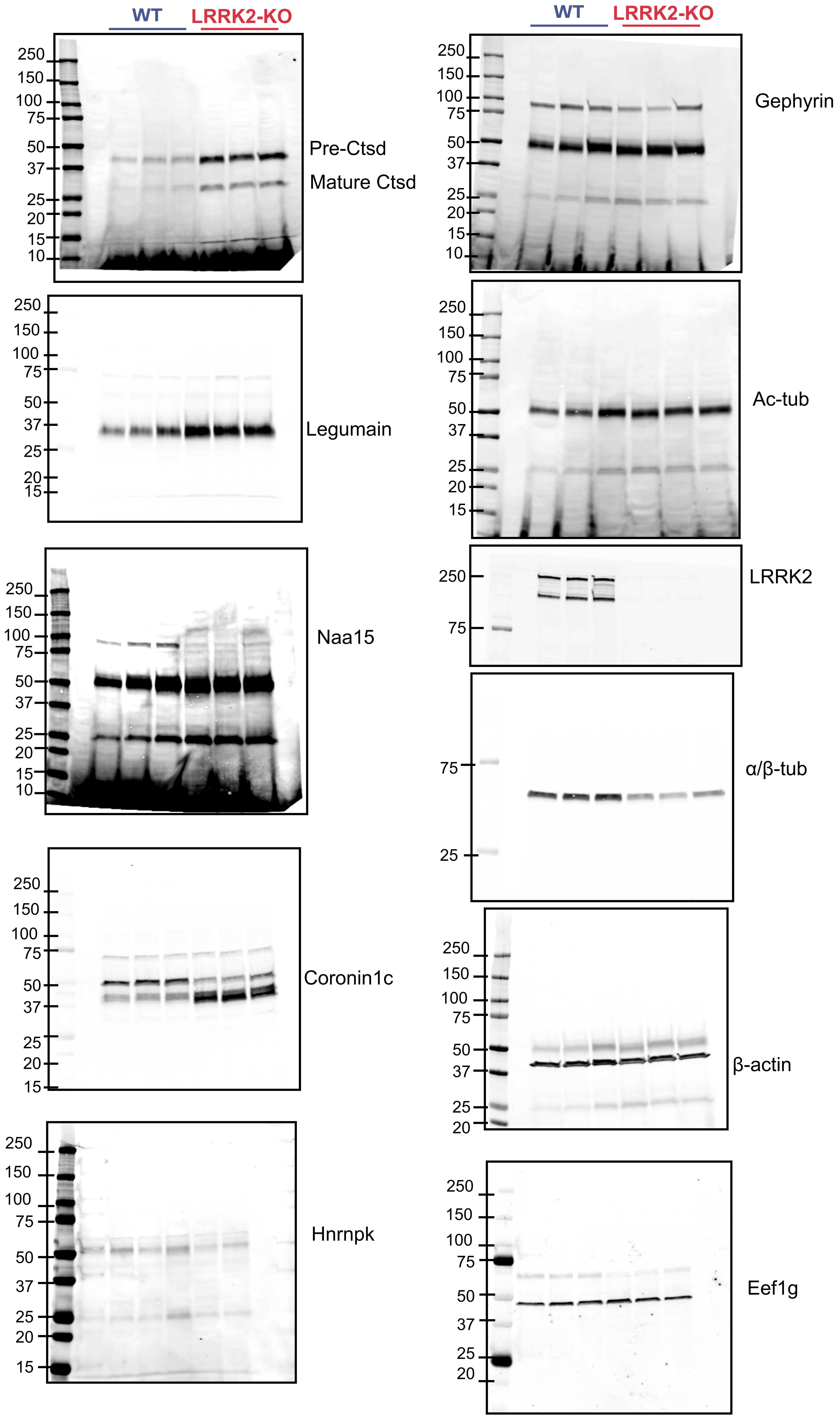

Uncropped immunoblots from kidney homogenates (12 month-old, n=4,5)

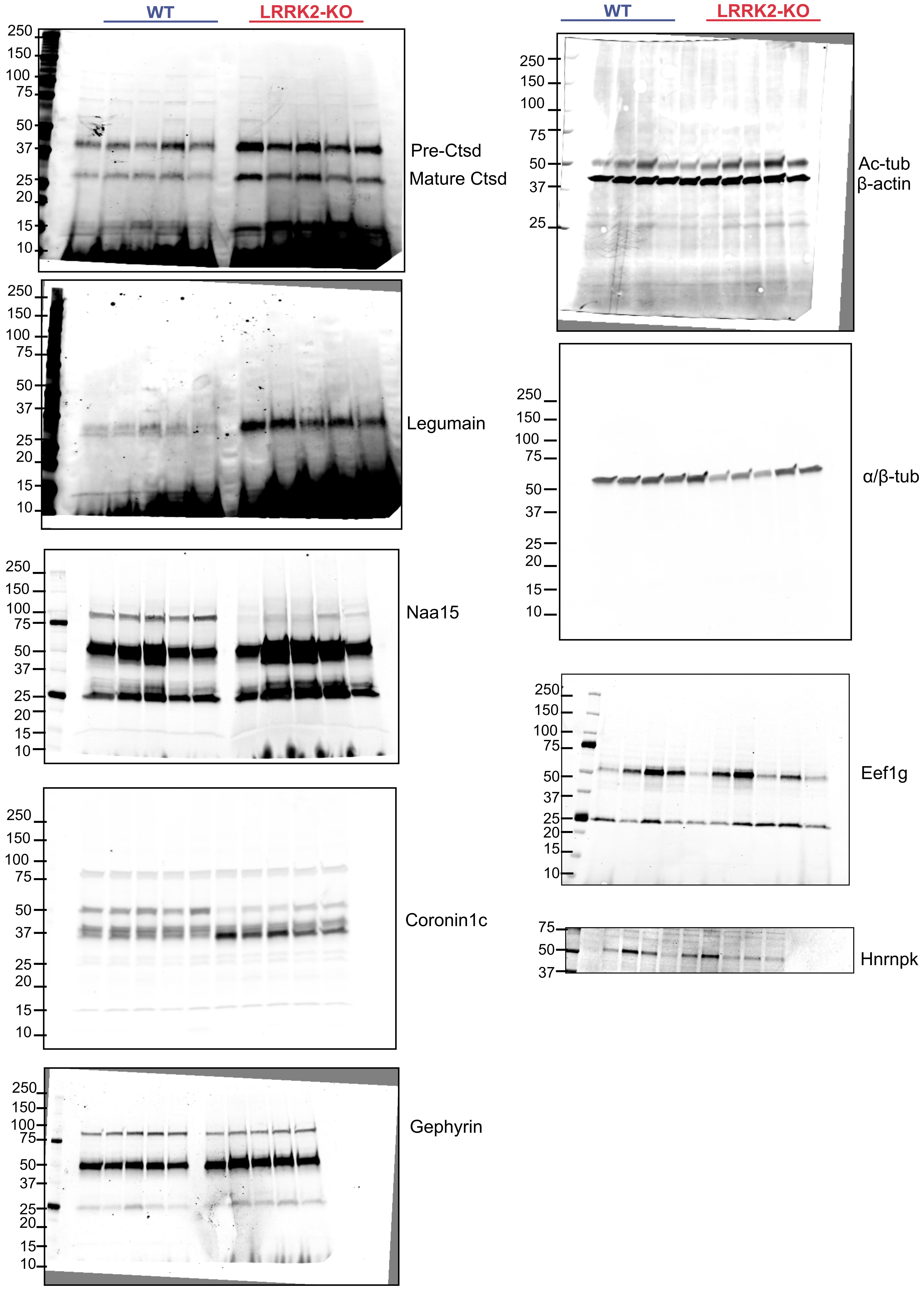

Uncropped immunoblots from kidney primary cells S6

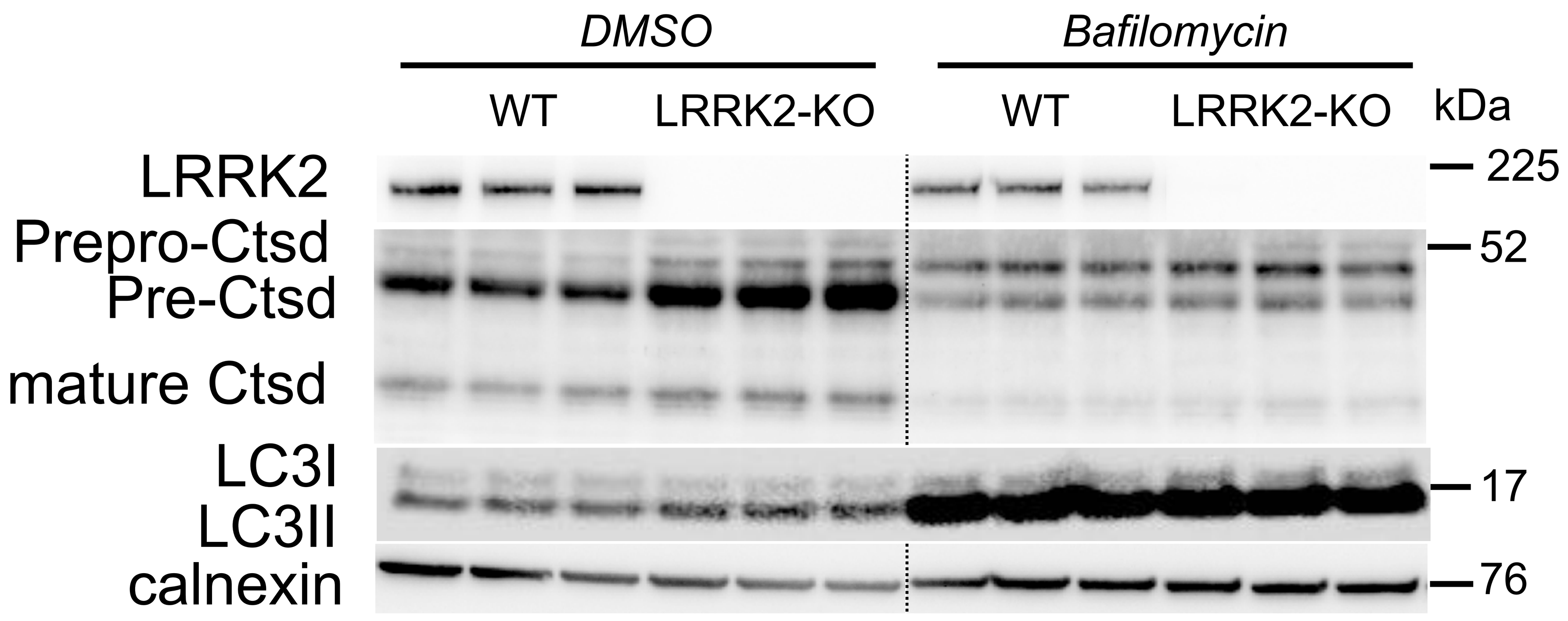

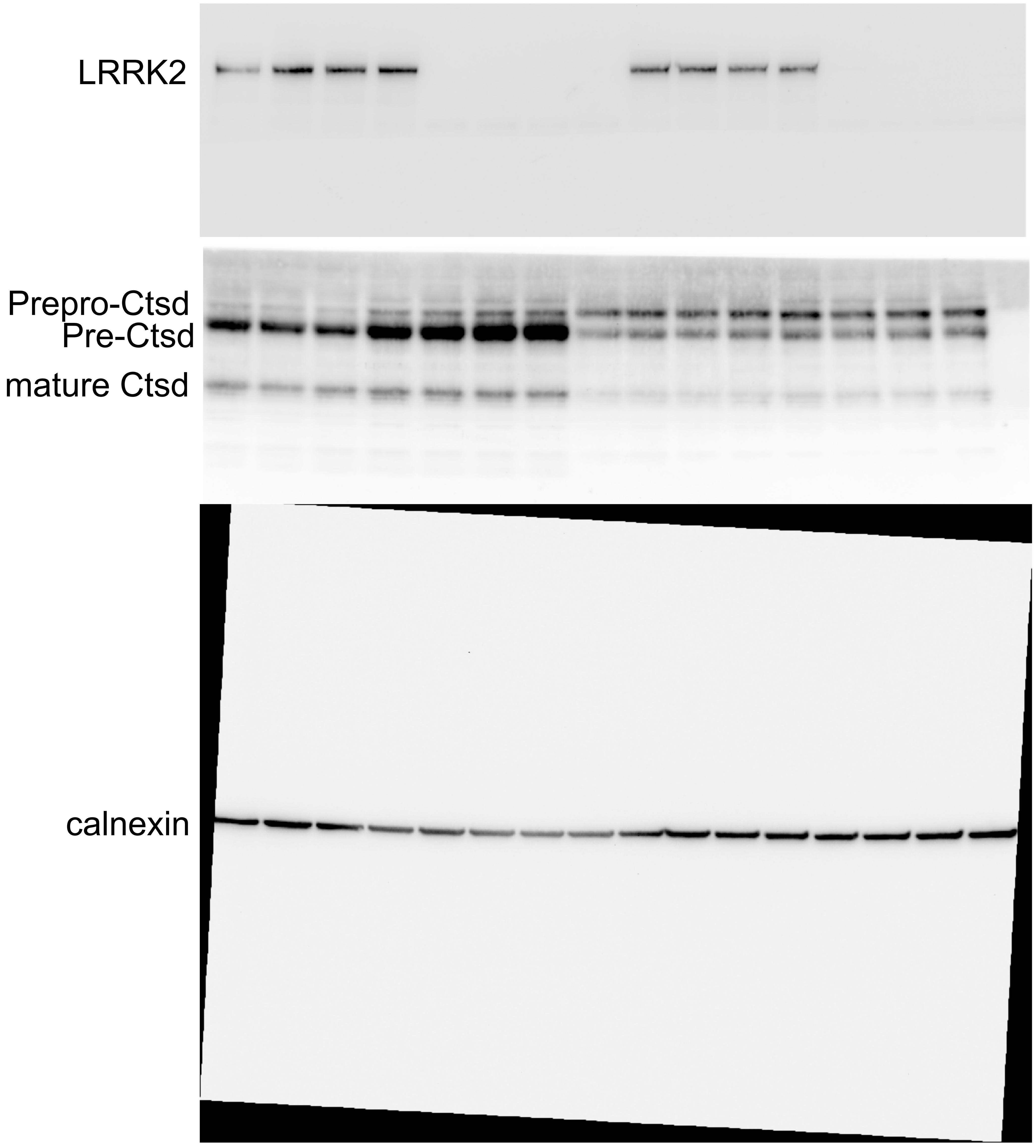

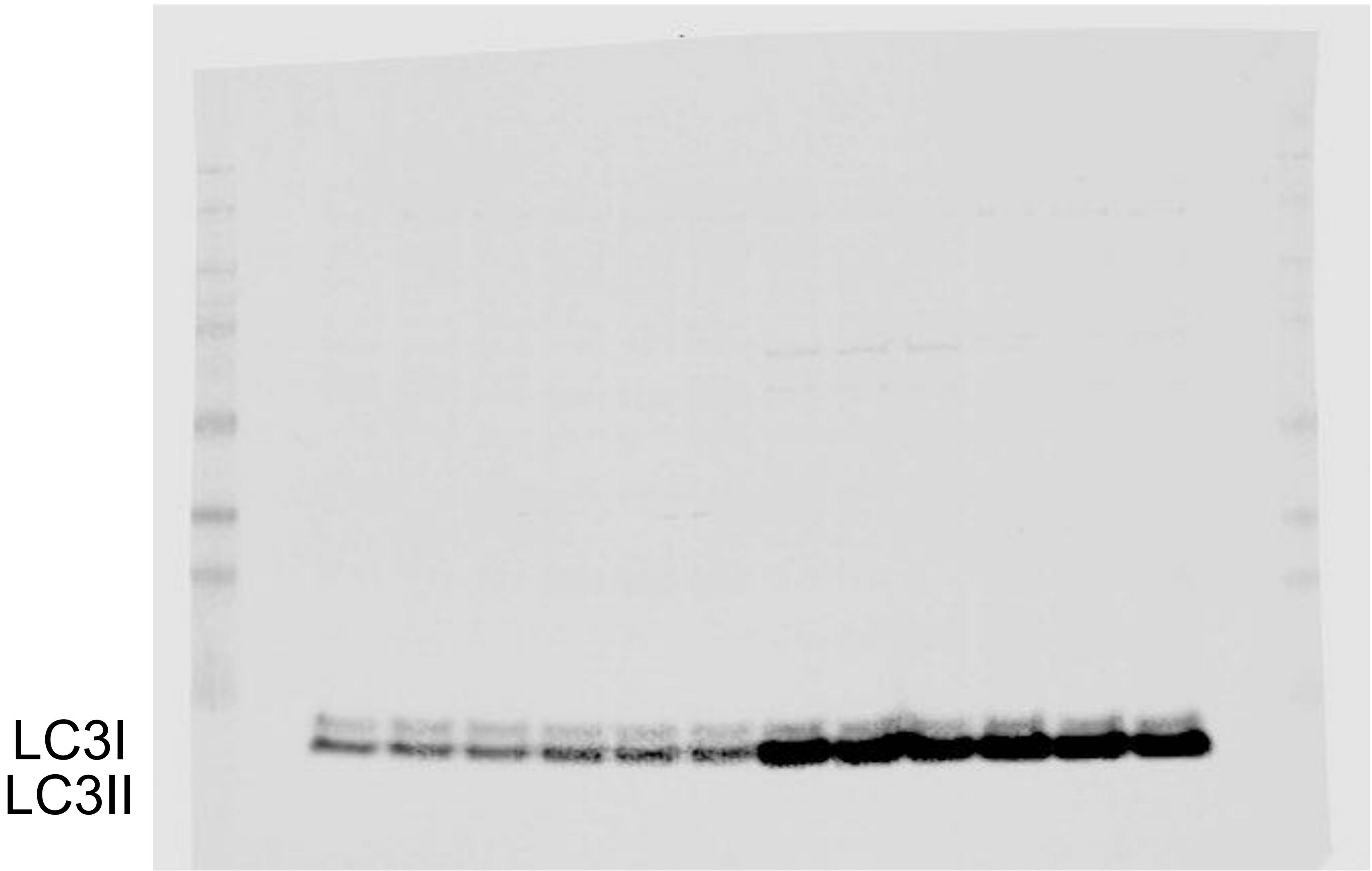

Supplement: Supplementary Data [file ddy232_supp.zip › Figures Pellegrini et al._HMG_revisions SUPPLEMENTARY DATA.pdf]
